# The role of seasonality in shaping the interactions of honeybees with other taxa

**DOI:** 10.1002/ece3.10580

**Published:** 2023-10-09

**Authors:** Helena Wirta, Mirkka Jones, Pablo Peña‐Aguilera, Camilo Chacón‐Duque, Eero Vesterinen, Otso Ovaskainen, Nerea Abrego, Tomas Roslin

**Affiliations:** ^1^ Department of Agricultural Sciences University of Helsinki Helsinki Finland; ^2^ Organismal and Evolutionary Biology Research Programme, Faculty of Biological and Environmental Sciences University of Helsinki Helsinki Finland; ^3^ Helsinki Institute of Life Science University of Helsinki Helsinki Finland; ^4^ Department of Ecology Swedish University of Agricultural Sciences Uppsala Sweden; ^5^ Centre for Palaeogenetics Stockholm Sweden; ^6^ Department of Archaeology and Classical Studies Stockholm University Stockholm Sweden; ^7^ Department of Biology University of Turku Turku Finland; ^8^ Department of Biological and Environmental Science University of Jyväskylä Jyväskylä Finland; ^9^ Department of Biology, Centre for Biodiversity Dynamics Norwegian University of Science and Technology Trondheim Norway

**Keywords:** *Apis mellifera*, Eltonian niche, honey, joint species distribution modeling, management, microbe, plant, whole genome sequencing

## Abstract

The Eltonian niche of a species is defined as its set of interactions with other taxa. How this set varies with biotic, abiotic and human influences is a core question of modern ecology. In seasonal environments, the realized Eltonian niche is likely to vary due to periodic changes in the occurrence and abundance of interaction partners and changes in species behavior and preferences. Also, human management decisions may leave strong imprints on species interactions. To compare the impact of seasonality to that of management effects, honeybees provide an excellent model system. Based on DNA traces of interaction partners archived in honey, we can infer honeybee interactions with floral resources and microbes in the surrounding habitats, their hives, and themselves. Here, we resolved seasonal and management‐based impacts on honeybee interactions by sampling beehives repeatedly during the honey‐storing period of honeybees in Finland. We then use a genome‐skimming approach to identify the taxonomic contents of the DNA in the samples. To compare the effects of the season to the effects of location, management, and the colony itself in shaping honeybee interactions, we used joint species distribution modeling. We found that honeybee interactions with other taxa varied greatly among taxonomic and functional groups. Against a backdrop of wide variation in the interactions documented in the DNA content of honey from bees from different hives, regions, and beekeepers, the imprint of the season remained relatively small. Overall, a honey‐based approach offers unique insights into seasonal variation in the identity and abundance of interaction partners among honeybees. During the summer, the availability and use of different interaction partners changed substantially, but hive‐ and taxon‐specific patterns were largely idiosyncratic as modified by hive management. Thus, the beekeeper and colony identity are as important determinants of the honeybee's realized Eltonian niche as is seasonality.

## Graphical abstract

Seasonality shapes the interactions of species both compositionally and functionally. We assessed honeybees' interactions with other species by identifying taxa bees have interacted with from DNA traces in honey. While the interactions varied both in identity and in functionality of the taxa, based on joint species distribution modeling, the time of the season had no larger impact than the location or management on the interactions of honeybees with other taxa.

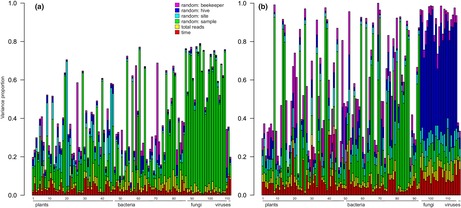

## 1 INTRODUCTION

The ecological niche of a species can be characterized from two perspectives: as the species' response to abiotic conditions (the Grinnellian niche; Grinnell, 1917; Whittaker et al., 1973) and as its interactions with other taxa in the surrounding community (the Eltonian niche; Elton, 1927). Over the past few decades, there has been a significant interest focused on characterizing species' Grinnellian niches due to changes in global abiotic conditions. However, the Eltonian niche is as important as the Grinnellian niche to be understood (see, e.g., Wirta et al., 2022), as environmental effects on both aspects of the niche are equally likely (Gravel et al., 2019; Pellissier et al., 2018). Thus, we should further our understanding of how external impacts shape community dynamics and ecological interaction networks, namely the Eltonian niche (Gravel et al., 2019).

In seasonal environments, the realized Eltonian niche set is likely to vary across the season, as driven by periodic changes in the occurrence and abundance of interaction partners and by changes in species behavior. Seasonality refers to major changes in a species' environment that are predictably repeated each year. How species' interactions are influenced by seasonal cycles has been the focus of intense research, in particular in the context of changing species' phenologies (e.g., Ekholm et al., 2019; Kešnerová et al., 2020; Rabeling et al., 2019).

Species inhabiting seasonally fluctuating environments experience variations in the intensity of their interactions, which are influenced by the changing seasons. In other words, seasonality is likely to shape different dimensions of the realized Eltonian niche differently, where some interactions are strongly affected whereas others are weakly affected or remain unaffected. On one end of the spectrum, certain interaction partners are only accessible during specific time windows, dictated by their own phenological patterns. This leads to a significant turnover in interaction partners over time. On the opposite end, another group of interaction partners remains active consistently through different seasons, resulting in minimal turnover in interactions. Nonetheless, it's worth noting that the impact of seasonality on species interactions has traditionally been examined for only a limited subset of interacting taxa at any given time. (e.g., Bauer & Hoye, 2014; Hutchison et al., 2020; Rasmussen et al., 2013, 2014; Rudolf, 2019).

Beyond seasonal effects, human management decisions may leave strong imprints on species interactions and therefore, on the realized Eltonian niches. By affecting the availability of resource species across landscapes, humans may strongly affect the set of realized interactions (Kortsch et al., 2023). For domesticated or half‐domesticated species, these effects will be most pronounced, as the human actor will affect both the focal species and which species it interacts with by actively altering, for example, its access to resources and its pathogen load.

A challenge for exploring the wholesale seasonal and anthropogenic drivers of the Eltonian niche is the complexity of resolving large sets of interactions in empirical systems. Here, the honeybee, *Apis mellifera*, offers a unique study system for assessing seasonal and other effects on the realized Eltonian niche (Wirta et al., 2022). Honeybees have been inserted by humans in environments characterized by different seasonality and different management practices throughout the world. Importantly, these interactions can be reconstructed from DNA traces left in honey (Bovo et al., 2018, 2020; Cirtwill et al., 2022; Galanis et al., 2022; Leponiemi et al., 2023). Such studies to date have shown that honeybees interact with a multitude of other taxa, most importantly flowering plants, but also microbes (Aizenberg‐Gershtein et al., 2013; Engel et al., 2016; Moran, 2015; Wirta et al., 2022).

While interactions between honeybees and plants tend to be mutualistic in nature, interactions between bees and microbes can be either pathogenic, mutualistic, or neutral in nature (Engel et al., 2016; Morse, 1994). As an example of a pathogenic interaction, the interaction of honeybees with the bacterium *Paenibacillus larvae* will cause severe disease in honeybees. In contrast, interactions of honeybees with *Snodgrassella alvi* or *Gilliamella apicola* can be described as mutualistic since these bacteria live in the honeybee's gut, sustaining the honeybee's health (Fünfhaus et al., 2018; Raymann & Moran, 2018).

The strongest effects of seasonality on honeybee niches will likely occur at high latitudes, where seasonal environmental changes are most pronounced. Here as everywhere else, bees will encounter a wide range of floral resources (Lehmuskallio & Lehmuskallio, 2006; Ruottinen et al., 2003; Salonen et al., 2009), but also interact with a range of microbes, including those present on flowers (Jones et al., 2018). Both microbes living in the hive and bee pathogens have been found to change seasonally (Donkersley et al., 2018; Runckel et al., 2011), possibly following the phenologies of different plant species. Thus, seasonality is likely to affect both mutualistic and antagonistic interactions between bees and other taxa.

Apart from seasonal effects on the honeybees, their Eltonian niche is likely to be shaped by anthropogenic factors with an impact on how the colony explores and utilizes its environment. Of particular interest are management practices, including measures of disease control, overwintering, and hive size. These effects can be captured by the identity of the beekeeper, who will apply similar methods to their hives (Morse, 1975, 1994; Ruottinen et al., 2003). However, even with similar management practices, individual hives sustained by an individual beekeeper will also differ from each other. This is due to differences in foraging behavior, foraging capacity, and susceptibility to diseases of the individual colony (Wray et al., 2011). Such aspects are strongly affected by the characteristics and health of the queen, shaping the performance of the colony and further modifying its behavior (Amiri et al., 2017). The effects of these factors can thus be captured by the identity of the hive. Additionally, the specific environment in which the hive is placed also affects the behavior of the colony.

In this paper, we use honey samples from Finland to compare the role of seasonality to that of management in determining the interactions of honeybees with other taxa (plants, bacteria, fungi, and viruses). For this purpose, we draw on a genome‐skimming approach to the DNA traces stored in the honey. To resolve temporal variation in the interaction records of honey, we sampled hives repeatedly during the honey‐storing period of honeybees and asked the following questions:

1. How does time of the season compare to the geographical location (site), management practices (beekeeper), and colony identity (hive) in terms of its influence on the taxa which honeybees encounter and interact with?
2. How does the use of flowering plants by honeybees change during the main flowering season (i.e., summer)?
3. How do the interactions between honeybees and microbes change during the summer, and how do patterns differ between different taxonomic and functional groups of microbes?
4. Will co‐occurrence patterns among taxa detected in temporally‐resolved honey samples suggest interactions or phenological associations among the taxa themselves?

## 2 MATERIALS AND METHODS

### 2.1 Seasonality in North‐European honeybee resources

In northern Europe, bees can actively interact with organisms outside their hive for about 6 months (Benno Meyer‐Rochow, 2008; Ruottinen et al., 2003). Pollen foraging typically starts in March or April, with the colony reaching its maximum size in May and June. From mid‐June to mid‐August, the bees work on storing honey, and by late‐August, the colony begins preparing for overwintering by producing the last workers of the year (Ruottinen et al., 2003). During this 6‐month period, the bees will interact with a range of flowering plants, each with its own phenology. Floral resources are typically most abundant in late‐June and July, when both early and late summer flowering species are in bloom simultaneously. This concerns both the species richness of flowering plants and their floral abundance. Of the typical plants used by honeybees in Finland, willows (*Salix* spp.) and dandelion (*Taraxacum* spp.) begin to bloom in May, then rape seed (*Brassica* spp.), raspberry (*Rubus idaeus*), clovers (*Trifolium* spp.), and fireweed (*Epilobium angustifolium*) flower from June onwards, and thistles (*Cirsium* spp.) and heather (*Calluna vulgaris*) begin their flowering only later in July (Benno Meyer‐Rochow, 2008; Lehmuskallio & Lehmuskallio, 2006; Salonen et al., 2009).

### 2.2 Using honey as an archive of interaction partners

A large proportion of taxa that honeybees encounter or interact with can be found in and identified from honey, where DNA traces of these taxa tend to be well preserved. By identifying the DNA found in honey, one can thus tell what other taxa honeybees have encountered or interacted with, especially for their interactions with microbes and plants (Bovo et al., 2020; Wirta et al., 2022). Adding to the information value of honey, nectar is spread into open combs for drying, and bees add enzymatic secretions during the processing of nectar into honey (Crane, 1979), and these processing stages would allow DNA present in any form within the hive to enter the nectar, turning it into honey. In practice, recently produced honey can be distinguished by its looks and position: on the honey frames of a hive, this fresh honey sits next to honey still uncovered by wax, and part of the combs are yet to receive a full wax cover. This new honey conveys a sample of the honeybees' interactions during the last week, corresponding to the time during which this nectar has been collected and processed into honey by the bees. In general, the time taken by nectar to ripen into honey tends to vary from 3 to 7 days, depending on weather, colony strength, and nectar availability (Crane, 1979; Morse, 1975, 1994).

Overall, the many processing stages involved in converting nectar to honey, the repeated manipulation of the nectar by the bee, and the time spent drying in open combs allow DNA present in multiple forms within the hive to enter the nectar. Thus, the honey of a beehive offers a well‐preserved record of recent interaction partners of its bees. However, it's crucial to note that not all DNA from interactions is carried back to the hive, and consequently, some of it does not become a part of the honey. For instance, when honeybees are preyed upon, the DNA of the predator is not included in the honey, leading to the undetected nature of these interactions.

### 2.3 Sampling

**FIGURE 1:**
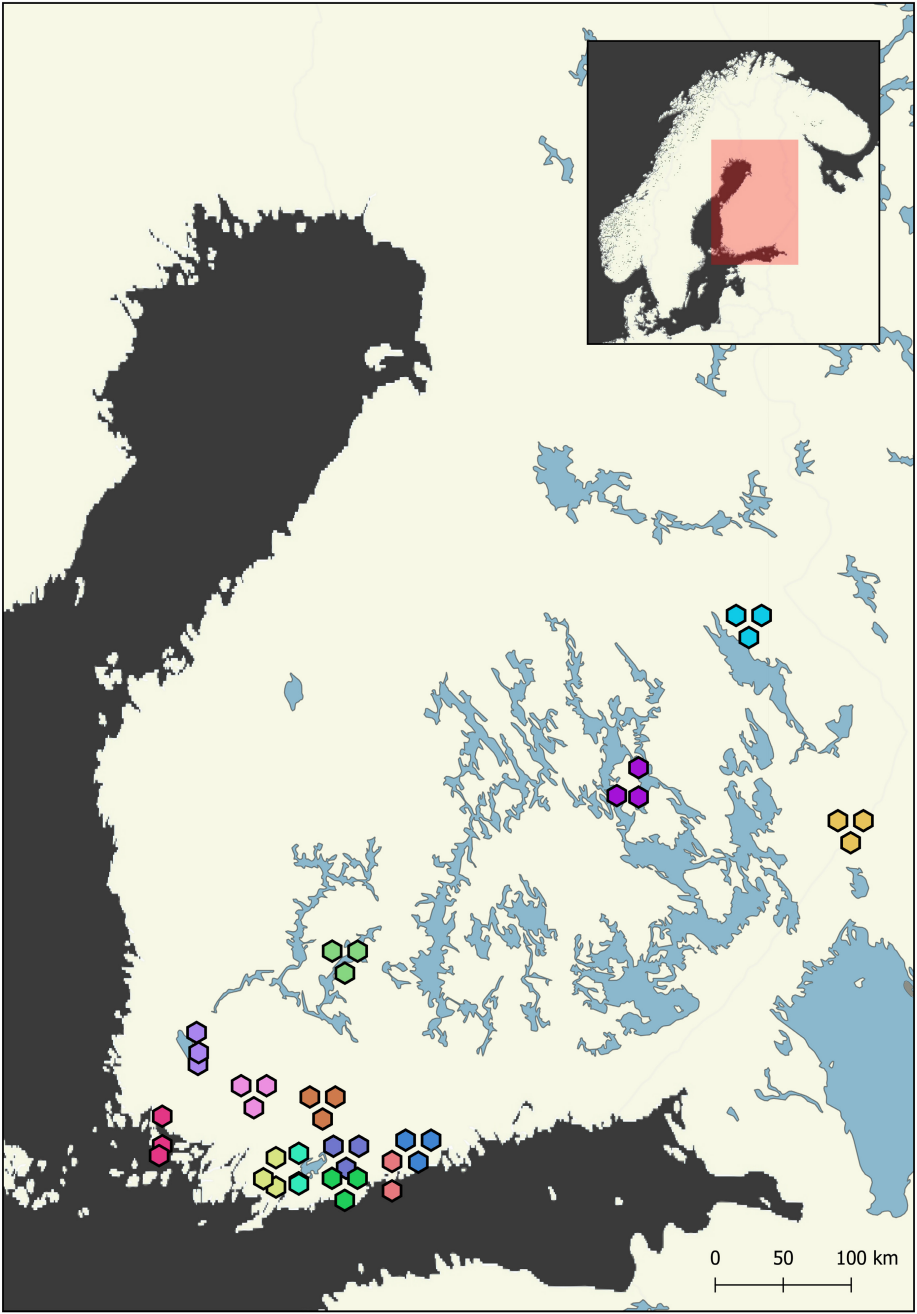
Locations of beehives sampled for honey in Finland, colored by beekeeper identity (with 2–3 hives sampled per beekeeper). Note that some beekeepers had hives at more than one site. To resolve overlapping sites, the locations of hives have been slightly jittered in both the horizontal and vertical planes.

To characterize seasonal variation in the microbial and floral interaction partners of honeybees, we obtained honey samples directly from beehives belonging to 14 Finnish beekeepers (Figure 1). Each beekeeper selected two or three of their hives, totaling 41 hives for the study. From each hive, honey was collected at three time points during the season, before the final harvest of all honey from the hives. Thus, samples were obtained from late‐June to mid‐August. All beekeepers were asked to sample their hives during the same weeks, corresponding to the 22nd to 28th of June, the 13th to 19th of July, and the 3rd to 9th of August of 2020. To ensure that the honey sample represented the specified time of the season, the beekeepers were instructed to collect only honey newly covered by wax. To obtain uncontaminated samples, we provided the beekeepers with DNA‐free sampling equipment. To ensure that the sample was representative of the variety of nectar recently collected, a spoonful of honey from three different frames was combined in each sample. Additionally, we also obtained a sample of honey from the end of season total yield of each beekeeper. This sample was used to assess whether such a time‐aggregated sample will include all the information gathered from the separate time‐specific samples.

Due to a dry period in early July of 2020, there was a shortage of flowers in parts of the study area. Therefore, July samples could not be obtained for all hives. Furthermore, for some of the samples, not enough DNA could be extracted from the 20 g of honey. Thus, in total, we were able to sequence 115 samples from individual hives and 13 samples of compound, end of season honey as harvested by individual beekeepers.

### 2.4 Laboratory methods

To identify the taxonomic origin of DNA in honey samples from different parts of Finland, we used a PCR‐free metagenomic approach. Instead of metabarcoding, where single genes are amplified and sequenced in a sample using primers targeted to the specific gene region (e.g., Vesterinen et al., 2018), we utilized a genome‐skimming approach to sequence random fragments of each species’ genome present in a sample without any locus‐specific PCR (see, e.g., Coissac et al., 2016).

To prepare the samples for DNA extraction, two subsamples of 10 g were each diluted with 30 mL of DNA‐free water (double‐distilled “MQ‐water”). These subsamples were allowed to dissolve for 1 h at +60°C. To collect all the tissue material and to remove excess water, the subsamples were centrifuged for 60 min at 8000 g (Centrifuge 5810 R, Eppendorf). Most of the supernatant was discarded, and the pellets from the two subsamples were combined into a 2 mL tube. The tube contents were further centrifuged for 5 min at 11,000 g (Heraeus Pico 21 centrifuge, Thermo Scientific). The remaining supernatant was discarded, and the pellets were stored at −20°C until DNA extraction.

Total DNA was extracted from each sample with the DNeasy Plant Mini Kit (Qiagen) with the following modifications to the protocol: Initially, the pellet was resuspended in 400 μL of buffer AP1, and then 4 μL RNase, 4 μL proteinase K (20 mg/mL, Macherey‐Nagel), and one 3 mm tungsten carbide bead was added to each sample tube. The sample was then disrupted for 2 × 2 min 30 Hz (Mixer Mill MM 400, Retsch). DNA extraction then followed the protocol, except that the QIAshredder column step was omitted to avoid DNA loss. All laboratory steps were done in a laminar hood wiped with ethanol and cleaned of DNA with 1 h of UV light every night. We only used DNA‐free tubes, pipette tips, and PCR plates, as well as DNA‐free water.

DNA quantity was measured with a Qubit 4 fluorometer (Thermo Fisher Scientific). For preparing the sequencing library, the samples were diluted to a concentration of 1 ng/μL. Samples with DNA concentrations <1 ng/μL were not diluted. The quality and quantity of DNA in each sample were measured with genomic DNA Tapestation and D500 HS Tapestation, before the preparation of the library. The Nextera XT transposome, provided with the Illumina Nextera XT library Preparation Kit (Illumina, Inc.), was used to fragment the DNA into 150‐bp‐long pieces and to tag the DNA with adapter sequences, following the Nextera XT Protocol. After this, an indexing PCR to anneal sample‐specific indexes to the DNA fragments was run, and the indexing PCR products were cleaned. The sample‐specific libraries were normalized to the same quantity, after which they were combined into the pooled library to be sequenced. All the steps to prepare the sequencing library from the total DNA followed the Nextera XT Protocol (Illumina Inc, 2019). The library was then sequenced in an Illumina NovaSeq6000 S4 flow cell, using 80% of one (out of four) flow cell lane, equaling 20% of the total sequencing capacity of the run. All sequencing was performed by the Functional Genomics Unit at the University of Helsinki, Finland. To detect possible contamination, we sequenced a DNA extraction blank control in the same way.

### 2.5 Bioinformatic processing

To remove any low‐quality bases from the start and end of reads and the Illumina adapter sequences, the raw reads were trimmed using Trimmomatic version 0.39 (Bolger et al., 2014) with the ILLUMINACLIP adapter‐clipping settings “adapters.fa: 2:30:10 LEADING:3 TRAILING:3 SLIDINGWINDOW:4:15 MINLEN:50”. To assemble trimmed reads into de novo scaffolds, we applied different *k*‐mer lengths [*k*‐mer = 21, 33, 55, 77, 99, and 121; following Nurk et al. (2017)] using the SPAdes assembly toolkit version 3.15.0 (Bankevich et al., 2012; https://github.com/ablab/spades) with the—meta flag (recommended for metagenomic data sets). To reduce heterozygosity, we then applied the Redundans pipeline (Pryszcz & Gabaldón, 2016) to the assembled scaffolds, with default values of identity 0.51 and overlap 0.8, and aligning all reads (align subset of reads with a limit value of 1). The reduced scaffolds were annotated to NCBI TaxIDs using BLASTN searches against the NCBI non‐redundant nucleotide database (nt) database (November–December 2021), keeping one aligned sequence per scaffold (max_target_seqs 1), saving only the best alignment for each query‐subject pair (max_hsps 1), and with an *E*‐value less than 1 × e^−25^. To map all the original trimmed and corrected sequences to the taxonomically annotated reference scaffolds, BWA MEM (Li & Durbin, 2009, 2010) was used, and the results were sorted into bam format files containing sample, sequence, and mapped read data with SAMtools (Li et al., 2009). For each assembly, the associated statistics at four taxonomic ranks (phylum, family, genus, and species) were generated with Blobtools (Laetsch et al., 2017) based on the BLASTn similarity search results.

To further filter all reads, with the intent of removing potentially misassigned reads and false positives due to tag jumping or contamination, we followed a conservative approach (following e.g., Alberdi et al., 2012; Lee et al., 2018). As a small number of reads representing a limited number of taxa were found in the control sample, we subtracted these reads from the read numbers of the corresponding taxa in the honey samples. As a final filtering step aimed at removing extremely rare and/or spurious reads, we calculated the mean relative read abundance (RRA hereafter; Deagle et al., 2019) of taxa (here genera) within samples and removed any taxa and reads assigned to taxa with a sample‐specific RRA of <0.001%. For the analyses, we only included genera with ≥0.01% mean RRA across the samples.

### 2.6 Occurrence of taxa and relative read abundances

In analyses based on RRA, a strong increase in the abundance of any taxon will, per necessity, be reflected in a reduction in the proportional representation of other taxa. Three genera dominated some samples: *Apilactobacillus* (*A. kunkeii*), *Zygosaccharomyces* (*Z. rougii*), and the virus *Apis mellifera* filamentous virus (AmFV; see Text S1 and Figure S1). For some of the samples, these taxa accounted for most reads (up to 85.8%, 91.2%, and 99.1% for *A. kunkeii*, *Z. rougii*, and AmFV, respectively). Thus, to restrict the impact of these taxa on patterns in other taxa, we also calculated RRA after omitting all reads assigned to the three dominant taxa identified above. In the analyses, we used the presence–absence data of all taxa (with mean RRA across samples ≥0.01%), but for abundance data, we omitted the three taxa with high yet variable proportions (Text S1 and Figure S1).

The proportions of reads assigned to individual kingdoms of associated taxa (plant, bacterial, fungal, and viral genera) varied substantially between samples even after removing *Apilactobacillus*, *Zygosaccharomyces*, and AmFV (Figure S2). Thus, while we included both plants and microbes in the analyses, we also described the changes in plants and in microbes separately from each other (Figures S3–S5).

The sequencing of the samples by Illumina NovaSeq s4, with 80% of a flow cell lane, resulted in 3.72 billion reads passing the filter (this sequencing run included 140 samples, out of which 118 plus a negative control sample are part of this study). For the samples in this study, 2689.8 million reads passed the quality controls, averaging 23.0 million reads per sample. 85.8% of these reads were assigned to the genus level and thus retained for further analyses. In addition to plants, microbes, fungi, and viruses, we identified 11 animal genera in the samples, but these were not considered in the analyses.

### 2.7 Functional groups of taxa

To resolve taxa of different functional affinities and of different associations with honeybees, we classified the genera following Wirta et al. (2022). The literature used in assigning taxa to specific functional groups is shown in Table S1. When reads within a genus were primarily (>90%) assigned to a given species, we based the functional assignment of the genus on information associated with this specific species. When reads within a genus were assigned to multiple species, we assessed the function based on a species known to be associated with honeybees. Finally, in the case where reads were not assigned to any particular species, we assessed the function based on the general biology of the genus.

Plants were classified into two groups based on their nectar‐producing ability. Microbes closely associated with bees were classified as common bee gut microbes, as beehive microbes, or as bee pathogens. Microbes without any known association with the bees were classified as plant pathogens, as animal pathogens, or as microbes known to be beneficial or neutral for plants and animals. Those microbe genera, which were known to have multiple roles, were categorized according to their relationship with honeybees. For instance, bacteria in the genus *Lactobacillus* could be present in nectar, but some species of this genus are considered ubiquitous in honeybee guts, and thus we classified *Lactobacillus* as a bee gut microbe (Raymann & Moran, 2018; Vannette, 2020). When the functional attribute of a genus was uncertain, then the genus was classified as unknown.

### 2.8 Statistical modeling

To examine the strength and patterns of seasonal imprints on honeybee associations and to compare them to the impacts of the beekeeper, the site, the hive, and the sample itself, we applied the joint species distribution modeling framework of Hierarchical Modeling of Species Communities (HMSC; Ovaskainen & Abrego, 2020).

To account for the zero‐inflated nature of the data, we applied a hurdle modeling approach, modeling presence–absence with probit regression and abundance conditional on presence using a log‐normal model. As response data, we used a matrix of presence–absences of all genera in the presence–absence models and the matrix of log‐transformed RRA's in models of abundance conditional on presence (henceforth referred to as abundance models). Since taxa with a particularly low or high prevalence contain little information on the factors affecting their occurrence, we excluded genera that were present in less than 5% of the samples from both models. We note that while presence–absences and abundances were modeled separately, these two models were used simultaneously to make predictions (see below).

The explanatory part of the models was identical, as follows: As fixed effects, we included the sampling period (a categorical variable with three levels) and the log‐transformed number of reads per sample. The variable of log‐transformed number of reads accounts for technical variation in sequencing depth among samples. Namely, this variable is meant to capture the effect of varying sampling effort among samples due to variation in sequencing depth. To account for the structure of the study design, we included as explanatory random effects the site (*n* = 30), the hive (*n* = 41), the beekeeper (*n* = 14), and the sample (*n* = 115), of which the site was defined as a spatially explicit effect. We note that the sample‐level random effect was included not necessarily to account for the spatial structure of the data but to estimate the species‐to‐species association networks through latent variable modeling (Ovaskainen et al., 2016). To test whether different taxonomic groups respond differently to sampling time, we included broad taxonomic (plants, bacteria, fungi, and viruses) and functional groups (described above) as genus‐level trait variables.

The models were fitted with the R‐package Hmsc (Tikhonov et al., 2020), assuming the default prior distributions (see Ovaskainen & Abrego, 2020, pp. 184–216). We sampled the posterior distribution with four Markov Chain Monte Carlo (MCMC) chains, each of which was run for 375,000 iterations, of which the first 125,000 were removed as burn‐in. The chains were thinned by 1000 to yield 250 posterior samples per chain and 1000 posterior samples in total. We examined MCMC convergence as a function of the potential scale reduction factors (Gelman & Rubin, 1992) of the model parameters.

The explanatory and predictive powers of the presence–absence models were examined through the metrics of Tjur's *R* ^2^ (Tjur, 2009) and the Area Under the Curve (AUC; Fielding & Bell, 1997). For the abundance models, we used the *R* ^2^ of the linear model (Ovaskainen & Abrego, 2020). To compute explanatory power, we made model predictions based on models fitted to all the data. To compute predictive power, we performed twofold cross‐validation, in which the substrate units were assigned randomly to twofolds, and predictions for each fold were based on a model fitted to the data on the other fold. To quantify what portion of the explained variance was attributed to each of the explanatory factors included in the models, we applied a variance partition approach. We then used the fitted models to build predictions on the responses of the genera to the season. To do so, we used the fixed effect part of the model only and predicted for each genus its occurrence probability for each of the three time points. We repeated the prediction for the 1000 samples of the posterior distribution to compute the posterior probability by which the genus had a higher occurrence probability in late season (August) than early season (June). We further converted the genus responses to the season to a temporal co‐occurrence matrix Ω, with the element corresponding to genus pair ($j_{1},j_{2}$) computed as $\Omega_{j_{1},j_{2}}=\beta_{T2,j_{1}}\beta_{T2,j_{2}}+\beta_{T3,j_{1}}\beta_{T3,j_{2}}$, where $\beta_{T2,j}$ and $\beta_{T3,j}$ are the genus responses to time points T2 (July) and T3 (August), with time point T1 (June) being set as the reference level. To examine the level of statistical support by which a given genus pair co‐occurs at the same time, we computed the posterior distribution of Ω and then evaluated the posterior probabilities by which each matrix element was positive or negative.

## 3 RESULTS

Overall, we detected a total of 49 plant genera, 45 bacterial genera, 23 fungal genera, and three viral genus‐level groups with a mean relative read abundance (RRA) exceeding 0.01%. The proportions of reads assigned to different kingdoms (plants, bacteria, fungi, and viruses) varied considerably between samples (Figure S2). Per sample, the average proportions of plants, bacteria, fungi, and viruses were 51%, 38%, 7%, and 3%, respectively.

### 3.1 Model fit statistics

**FIGURE 2:**
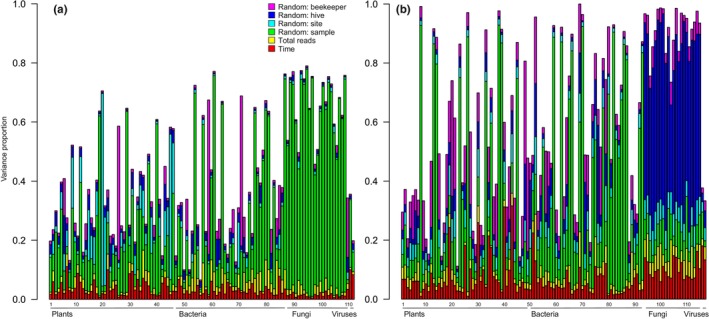
Taxon‐specific proportions of total variance attributed to each fixed (time and total reads) and random effect (beekeeper, hive, site, and sample) in the models of (a) presence–absence and (b) abundance conditional on presence. Within each taxonomic group, the genera (bars) are ordered alphabetically, with genus names shown in Tables S2 and S3.

The fitted joint species distribution models showed high explanatory power both for the presence–absence (Tjur's *R* ^2^ = 0.42 and AUC = 0.93) and abundance conditional on presence (*R* ^2^ = 0.64) models (Table 1 and Figure 2a). Nonetheless, the explanatory power varied widely among genera as well as among taxonomic and functional groups. Among taxonomic groups, the explanatory power was highest for fungi, explaining 65% and 91% of the variation in the presence–absence and abundance models, respectively. Among functional groups, animal pathogens reached the highest explanatory power, explaining 69% and 92% of the variation.

**TABLE 1:** Mean percentages of variance explained by the fixed and random effects included in the models, and their summed total explanatory power quantified by Tjur *R* ^2^ for the presence–absence model and *R* ^2^ for the abundance conditional on presence model.

	Taxonomic groups					Functional groups							
	Total	Plants	Bacteria	Fungi	Viruses	Nectar producing	No nectar producing	Bee gut	Beehive	Bee pathogens	Plant pathogens	Neutral or positive	Animal pathogens
*Presence–absence*													
Time	3.2	3.9	3.1	1.4	9.2	3.3	7.3	3.3	3.9	3.5	2.2	2.2	2.0
Total_reads	2.9	2.1	4.2	2.1	0.9	1.8	4.0	2.8	3.3	3.6	3.5	2.3	4.2
Random: sample	26.3	15.8	21.0	58.0	14.7	16.0	14.2	11.6	26.9	29.6	46.2	26.1	61.1
Random: site	4.0	7.8	1.7	1.0	0.6	8.0	6.8	1.5	1.3	4.1	0.7	0.9	0.9
Random: hive	2.3	3.0	2.4	0.9	1.5	2.7	4.7	3.9	2.3	1.9	0.7	2.3	0.6
Random: beekeeper	3.2	2.6	4.8	1.5	0.8	2.8	2.0	2.3	5.9	2.9	0.7	21.1	0.5
Explanatory power Tjur *R* ^2^	42	35	37	65	28	35	39	25	44	46	54	55	69
*Abundance conditional on presence*													
Time	7.4	7.7	5.4	9.8	15.7	8.0	6.0	5.4	2.8	7.7	8.7	7.5	7.0
Total_reads	3.7	2.7	3.4	6.3	3.4	2.8	2.3	2.7	1.3	3.6	5.8	1.5	5.0
Random: sample	22.6	20.4	33.2	8.0	5.5	23.4	2.2	16.3	60.7	16.6	27.6	28.0	38.5
Random: site	5.3	4.8	5.0	7.0	4.0	5.0	3.5	4.8	2.5	6.7	5.4	6.5	4.8
Random: hive	14.2	3.8	5.7	55.1	4.4	4.1	2.3	5.2	3.2	21.1	32.7	13.9	33.4
Random: beekeeper	8.1	10.3	7.2	5.2	2.5	10.6	8.3	7.2	1.7	7.7	4.8	14.9	2.8
Explanatory power *R* ^2^	61	50	60	91	36	54	25	42	72	64	85	72	92

*Note*: On each row we report the average values over all the taxa modeled (column “total”) and per individual taxonomic and functional group (all other columns).

The predictive power was far lower than the explanatory power for both the presence–absence (Tjur's *R* ^2^ = 0.11 and AUC = 0.63) and abundance models (*R* ^2^ = 0.02). However, this result seems attributable to the fact that the sampling unit‐level random effects (i.e., sample level) accounted for a large part of the explained variation (22.3% for the presence–absence model and 22.6% for the abundance model). These random effects will contribute to the explanatory power but not to the predictive power of the models.

### 3.2 Seasonal effects on the interactions of honeybees

A variance partitioning among the fixed and random effects showed that the seasonal imprint explained, on average, 3.2% of the raw variance in the presence–absences and 7.4% in the abundances of the taxa. The strength of the imprint of the beekeeper, hive, and site on the occurrences of the interactions of honeybees was similar to that of time of the season (with the beekeeper, hive, and site explaining 3.2%, 2.3%, and 4.0% of the variance, respectively). However, the hive had a stronger effect on the abundances of the taxa honeybees interact with, explaining 14.2% of the variance.

The proportion of variance attributed to the time of the season varied greatly among taxa and among both taxonomic and functional groups (Table 1 and Figure 2). Among taxonomic groups, viruses were the most influenced and fungi the least influenced by the time of the season (both in terms of presence–absence and abundance). Among functional groups, the no‐nectar‐producing plant taxa were the most influenced by the time of the season. The amount of variation explained by the time of the season varied not only among taxonomic and functional groups but within groups as well (Figure 2). The occurrences and abundances of some taxa were well explained by the time of the season, whereas the occurrences and abundances of other taxa were totally unaffected by the time of the season. As examples, the occurrence of some plants was strongly impacted by the time of the season. For *Chamaenerion*, sampling time accounted for 11.1% of the variation in the presence–absence model, while for *Lactuca*, the time of the season accounted for only 0.7% of the variation (Table S2). In terms of abundances, *Taraxacum* was the genus most impacted by the time of the year (with time accounting for 31.6% of the variation), while *Medigaco* and *Cicer* fell at the opposite extreme (with time accounting for 1.0% of variance explained; Table S3). In regard to microbes, the time of the season impacted the occurrences of the two viral groups the most (accounting for 8.3% and 10.0% of their variation for the presence–absence model), while the time of the season had the least impact on the fungal genus *Histoplasma* (accounting for 0.5% of its variation; Table S2). For the abundances, the time of the season had a strong impact on the bacterial genus *Acinetobacter* (accounting for 22.4% of the variation), while the bacterial genera *Pantoea* and *Pectobacterium* were the least impacted by time (accounting for 1.2% and 1.5% of variation, respectively; Figure 2 and Table S3).

Furthermore, the temporal patterns of honeybee interactions with different plant genera differed strongly among hives. Some bee colonies, that is, honeybees from particular hives, used a similar set of plant genera throughout the summer, with only gradual changes in their relative proportions (Text S2). Other colonies shifted strongly to a particular plant genus, such as *Brassica*, from one time point to the other (Figure S3). The occurrences and relative abundances of microbes differed greatly among colonies and across time points (Figure S4). Curiously, for some colonies and samples, the microbe community was almost solely composed of fungi, with either no bacterial genera or only a few present at very low relative abundances (Text S2 and Figure S4). Overall, the compound samples collected at the end of the summer did not reveal the diversity of plants, fungi, and bacteria exposed by the time‐resolved samples (Text S3 and Figures S7 and S8).

**FIGURE 3:**
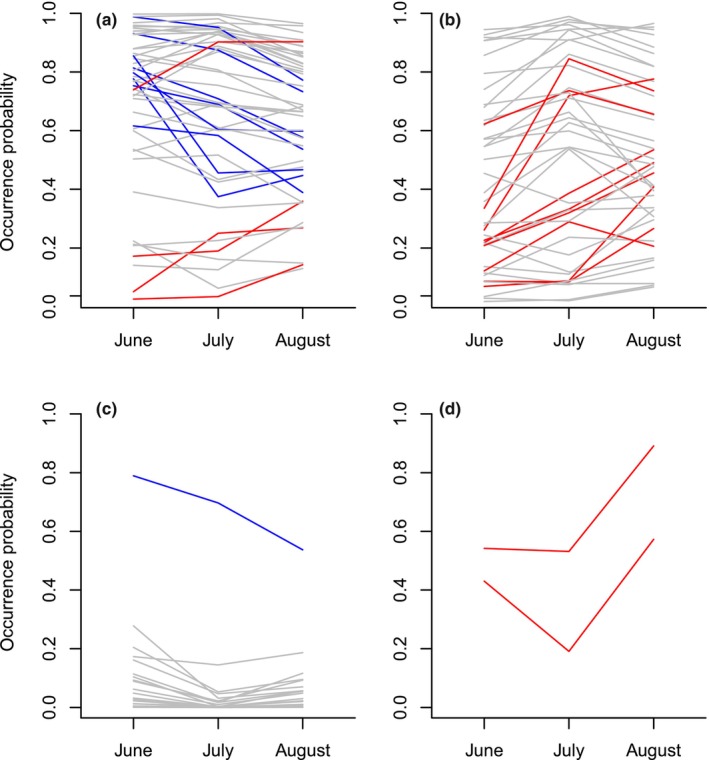
The predicted occurrence probability of each genus at the three times of the season for plants (a), bacteria (b), fungi (c), and viruses (d). Each line shows the posterior mean probability of a genus's occurrence in June, July, and August. Genera for which occurrence probability increases directionally over the season are shown in red, whereas genera for which occurrence probability decreases over the season are shown in blue (with at least 95% posterior probability for higher or lower, respectively, occurrence probability in August than in June).

The predicted occurrence probabilities of taxa belonging to different taxonomic groups varied throughout the season, with a minority of the genera showing a clear directional change over the season in any of the taxonomic groups (Figure 3). Plants showed the most variation in their predicted occurrence probabilities (Figure 3a). While the occurrence probabilities of most bacteria did not show statistically supported changes throughout the season, the taxa that did change showed an increase (Figure 3b). In the case of fungi, only a single taxon showed a decreasing trend during the season (Figure 3c). Two virus taxa showed a statistically supported increasing trend in their occurrence probabilities (Figure 3d). For the functional groups (Figures S5 and S6), for some of the nectar‐producing plants, the occurrence probability increased, whereas for non‐nectar‐producing plants, the occurrence probability decreased throughout the season (Figure S6a,b). Among microbial functional groups predicted to increase toward the end of the season, most were gut‐associated microbes and bee pathogens (Figure S6c,e). No plant pathogens (Figure S6f) nor any animal pathogens (Figure S6h) showed statistically supported changes over the season. Only a single hive‐associated microbe showed a decreasing trend (Figure S6d), whereas a single neutral microbe showed an increasing trend (Figure S6g) over the flowering season.

### 3.3 Co‐occurrences of genera through the season

**FIGURE 4:**
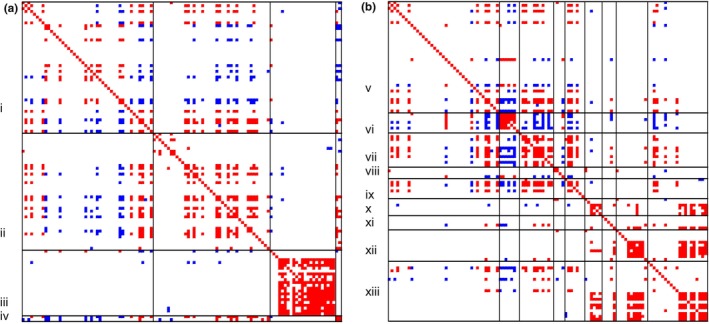
Seasonal co‐occurrences among the genera. Genus pairs that are more likely to occur at the same time point than expected by random (with at least 90% posterior probability) are shown in red, whereas species pairs that are less likely to occur at the same time point than expected by random (with at least 90% posterior probability) are shown in blue. In (a) the species are ordered based on their taxonomic group: plants (i), bacteria (ii), fungi (iii), and viruses (iv), whereas in (b), they are ordered based on their functional group: nectar‐producing plants (v), non‐nectar‐producing plants (vi), gut microbes (vii), hive microbes (viii), bee pathogens (ix), plant pathogens (x), neutral or positive microbes (xi), animal pathogens (xii), and unknown (xiii). In both panels, the species are ordered within each group according to decreasing prevalence.

The temporal genus‐to‐genus association matrix revealed that many of the taxa associated with honeybees co‐occurred either positively or negatively with each other during different time points (Figure 4). One group of temporally co‐occurring species was formed by non‐nectar plants, and another group by bee gut microbes and bee pathogen microbes. These two groups showed negative associations (Figure 4), as generated by non‐nectar plants thriving in the early season and bee gut and bee pathogen microbes thriving at the end of the season (Figure S6). As the occurrence of different nectar plants varied differently over the season (Figure S6), some of the nectar plants co‐occurred in time with non‐nectar plants, while others co‐occurred with bee gut and bee pathogen microbes. Plant pathogens and animal pathogens did not show any major patterns of co‐occurrence with the other functional groups, whereas genera within these groups showed patterns of positive co‐occurrence.

## 4 DISCUSSION

The honeybee offers unique insight into local and seasonal variation in the realized Eltonian niche of a semi‐domesticated species and into the relative strength of impacts on its niche. Based on interactions archived in honey, we can infer how the progression of the summer season changes the honeybees' interactions with floral resources and microbes in the surrounding habitats, in the hives, and within the bees themselves. We find that the seasonal patterns of honeybee‐associated taxa vary greatly among taxonomic and functional groups—and that against the backdrop of wide variation in the interactions documented in the DNA content of honey from bees from different hives, regions, and beekeepers, the imprint of season remained relatively small. Below, we will evaluate these patterns in further detail.

### 4.1 Changes in flower usage over the season

In terms of the occurrences of different plant genera in honey, our study reveals an imprint of season of similar strength to those of site, management, and colony identity. Thus, the overall use of different plant taxa does not vary systematically across the summer. In terms of the relative abundances of plant genera, the time of the season had a larger but still limited impact, accounting for 1.0%–31.6% of overall variation.

The limited imprint of season on the selection of plants contrasts with previous studies showing major variation in plant use by honeybees across the season. Working within the hyper‐diverse plant community of a botanical garden, Lowe et al. (2022) found major changes in floral use during a summer. Seasonal shifts in foraging choices have also been detected in agricultural environments, both in terms of the diversity and in amounts of pollen collected (Danner et al., 2017). Here, our results on relative abundances are more in line with previous results, showing a larger impact of the time of the season.

In terms of proportions of plant genera, we found that of nectar‐producing plants to be high throughout the summer, while non‐nectar‐producing plants were present in far lower proportions. This is only to be expected since honey is produced from nectar. The average occurrence probability of nectar‐producing plant genera increased from June to August, while that of non‐nectar‐producing genera decreased. Non‐nectar‐producing trees and grasses, such as *Picea* and *Pinus*, and *Hordeum* and *Triticum*, abundant in our study area, flower early in the summer (Lehmuskallio & Lehmuskallio, 2006; Peltonen‐Sainio et al., 2005). Thus, the observed decrease in their representation in honey over the season aligns well with their flowering phenology and thus with the likely frequency with which they are encountered by honeybees.

Variation in patterns of floral resource use by bees was not strongly impacted by location, neither in terms of occurrence nor relative abundances of DNA in honey. This pattern is consistent with previous results showing little impact of site in large scale on plant selection. Jones et al. (2021) found no significant effects of the source region on the composition of plant taxa in honey, whereas Danner et al. (2017) found no effect of different agricultural landscapes on the taxa chosen for pollen.

The identity of the beekeeper did have a major impact on the relative representation of plant taxa among honeybee interactions stored in honey. Here, the relatively large role of the beekeeper could partially arise from specific choices regarding where the hive is placed. To support the well‐being of the bee colony and to improve honey yields, any beekeeper is likely to locate their hives close to good nectar and pollen sources. Effects of proximity to, for example, flush‐flowering crop plants have indeed been found in earlier studies of floral choices (Danner et al., 2016, 2017), and the impact of the specific site, on a smaller scale, has been shown to impact flower choices (Leponiemi et al., 2023). In the current study, we did not directly assess the effects of crop proximity, but in evidence of the effect of mass‐flowering crops on the plant selection of bees, we did find abrupt changes in plant use by some of the colonies. In particular, some colonies exhibited quick shifts to using rapeseed oil (*Brassica*) during the flowering time of this crop. As foraging is both highly energy‐consuming and exposes the foragers to predation, any bee colony will clearly benefit from a foraging strategy aimed at maximizing nectar yields while minimizing foraging distances (Danner et al., 2016).

### 4.2 Spatiotemporal variation in the realized Eltonian niche of honeybees

Microbes form a major part of the taxonomic diversity with which honeybees interact. The gut microbes of honeybees consist of five ubiquitous taxa and a few other taxa dominating the gut microbiota of most bee individuals (Moran, 2015; Raymann & Moran, 2018). Five core taxa (*Lactobacillus* Firm‐4 and Firm‐5, *Snodgrassella alvi*, *Gilliamella apicola*, and *Bifidobacterium* spp. Raymann & Moran, 2018) were all detected in our honey samples. These bacteria are acquired through social activities within the colony, such as bees' contact with nurse bees, with the feces of other bees, and with the hive environment itself soon after the emergence of a bee (Engel et al., 2016; Kwong et al., 2017; Moran, 2015). Other microbes with which honeybees interact will mostly comprise taxa originating from the environment, such as microbes in food stores, on hive surfaces, and on the body surface of honeybees (Aizenberg‐Gershtein et al., 2013; Donkersley et al., 2018; Muñoz‐Colmenero et al., 2020). Thus, the overall honeybee microbiota can be seen as consisting of two rather distinct types: a stable, low‐diversity gut microbiota and a variable, highly diverse overall bee microbiota. The composition of this latter community will depend on the factors that influence the exposure of honeybees to different microbes in the environment.

In terms of microbe occurrences, we found a similar effect of season on microbes living in the honeybee gut as on other microbes with which the honeybees interact. Yet, in terms of relative abundances, the impacts of time were more pronounced for microbes living in the bee gut than for other microbes. For microbial genera in the bee gut, the occurrence probability generally increased toward August, while microbes typical of the beehive showed no directional temporal changes. These conflicting patterns can then be contrasted with previous findings showing the gut microbiota to be more stable than the overall honeybee microbiota (Corby‐Harris et al., 2014).

More generally, the time of the season and the characteristics of the region surrounding the hive have been previously found to influence the honeybee microbiota, but the patterns previously reported seem mixed. In one study, the microbiota of bees, including microbes in and on bee individuals, was found to change markedly with the time of the season (Almeida et al., 2023). In another study, the change was found to be very small (Subotic et al., 2019)—which the authors attributed to the high dominance of gut microbes in the overall microbiota of any bee individual (Subotic et al., 2019) and to the proposed stability of gut microbiota over time (Corby‐Harris et al., 2014). On the other hand, the species richness of bacteria related to beebread (i.e., stored pollen) has been shown to change across the seasons and to be at its lowest during the middle of the summer (Donkersley et al., 2018).

For pathogenic microbes in honeybees, our study predicted a slight increase from June to August. This pattern is in line with the small variation of pathogens documented in bees within the flowering season and with a larger variation across the entire year (Runckel et al., 2011). Among the fungal pathogens of honeybees, *Nosema ceranae* appears to be ubiquitous across hives and across the warm season (D'Alvise et al., 2019). *Nosema apis* was found to peak in abundance early in the summer and *N. ceranae* during the late summer, yet there were large changes in abundance in the winter months (Runckel et al., 2011). For the foulbroods (i.e., honeybee pathogens causing fatal brood infections), our results show that the occurrence of *Melissococcus* was strongly affected by the site, but not by the time of the season nor by the beekeeper. For *Paenibacillus* (the causative agent of American foulbrood), the relative abundance was mostly impacted by the site. This pattern matches findings from a German study, which showed foulbroods to be highly specific to individual geographical regions (D'Alvise et al., 2019).

For the majority of bacterial and fungal taxa, site identity accounted for only a small fraction of the explained variance in occurrences. Nonetheless, the impact of the site varied greatly among microbial groups. Features of the study area (including landscape characteristics and/or the dominant crop species) have been shown to shape the bee microbiota with varying strengths, with impacts ranging from mild (Almeida et al., 2023) to strong (Aizenberg‐Gershtein et al., 2013; Muñoz‐Colmenero et al., 2020; Subotic et al., 2019). Landscape attributes have also been shown to affect the gut microbiome (Jones et al., 2018). Thus, the overall impacts of the time of the season and the site on these microbes vary substantially. As part of these microbes have a major effect on bee health (Anderson et al., 2013; Engel et al., 2016; Hedtke et al., 2011), the observed variation largely adds to the challenges in predicting honeybee health and disease.

The role of the beekeeper was similar to that of time and site in defining both the occurrences and relative abundances of microbes. This imprint of the beekeeper can be ascribed to their role in the practical management of the hive. The beekeeper will, for example, decide where the hive is placed and how densities of the parasitic *Varroa* mite are controlled. The beekeeper will also decide on hive size to achieve a suitable temperature and storage space and feed the bees during natural food shortages. The impact of management appears to be particularly strong on the occurrences of two bacterial genera: *Entomoplasma* and *Mesoplasma*. These bacteria may play either a protective role against pathogens of plants and insects or a neutral role (Baby et al., 2018; Fünfhaus et al., 2018; Gasparich, 2014). Hence, management decisions with an impact on these microbes may not directly affect bee health but still affect the overall composition of the honey microbiota.

### 4.3 Interactions among taxa may shape whom honeybees interact with

Among hives managed by the same beekeeper, the identity of the individual colony had a surprisingly large added impact on microbe occurrences and relative abundances. The effect of the colony was at least as large as that of the beekeeper, and sometimes far larger. This effect was particularly pronounced for fungi and viruses. The relative abundance of fungi varied strongly among hives, with an average of over 55% of the raw variation in taxon‐specific relative abundances explained by the hive. Such patterns can likely be ascribed to the many factors that vary among colonies, such as the age and health of the queen—but also to individual infection events, first affecting the presence–absence of species and thereafter the growth of the microbe in the hive (cf. Figure 2).

Although our study falls short of establishing causality between the abundance of fungi and other taxa, the hives in which fungal taxa were common and abundant were characterized by a relatively high abundance of two common fungal bee pathogens: *Ascosphaera*, causing the brood disease chalkbrood, and *Aspergillus*, causing stonebrood (Evison & Jensen, 2018; Foley et al., 2014). High local abundances of these taxa could indicate a diseased colony. In many of the hives where fungi occur, there was a strikingly low relative abundance of bacteria. This could be due to characteristics of the fungi, as, for example, *Aspergillus penicillium* produces penicillin (Al‐Fakih et al., 2019), which may kill bacteria. Such a pattern urges further research on how microbes within the bee microbiome affect each other.

## 5 CONCLUSIONS

Characterization of the Eltonian niche of species remains a challenging task. To resolve systematic imprints on the realized Eltonian niche, we need to replicate sets of interactions across time and space. Our study provides a DNA‐based template for such endeavors, as we here use a genome‐skimming approach to identify the taxonomic contents of DNA in honey samples collected across time and space. This allowed us to resolve wide variation in the interactions archived in the DNA content of individual honey samples from different times of the seasons, hives, regions, and beekeepers, and wide variation in interaction patterns with individual taxa and functional groups. Overall, the variation resolved reveals just how dynamic the realized Eltonian niche of a species will be under the simultaneous impacts of both people and environment. Thus, to understand how species' are changing their roles with global change, and to understand how external impacts will shape the dynamics of ecological communities and interaction networks, we urgently need to resolve current impacts on both the Eltonian and Grinnellian niches of species. Our current study makes a start by resolving the impacts of season versus management on the realized Eltonian niche—but the same approach can clearly be extended to niche variation along any environmental or anthropogenic dimension.

## AUTHOR CONTRIBUTIONS

**Helena Wirta:** Conceptualization (lead); data curation (lead); formal analysis (supporting); funding acquisition (lead); investigation (lead); methodology (equal); project administration (lead); resources (supporting); visualization (supporting); writing – original draft (equal); writing – review and editing (lead). **Mirkka Jones:** Formal analysis (equal); methodology (supporting); validation (supporting); writing – review and editing (supporting). **Pablo Peña‐Aguilera:** Formal analysis (supporting); methodology (supporting); visualization (supporting); writing – review and editing (supporting). **Camilo Chacón‐Duque:** Formal analysis (supporting); methodology (supporting); resources (supporting); writing – review and editing (supporting). **Eero Vesterinen:** Methodology (supporting); resources (supporting); writing – review and editing (supporting). **Otso Ovaskainen:** Formal analysis (equal); methodology (supporting); visualization (supporting); writing – review and editing (supporting). **Nerea Abrego:** Formal analysis (equal); methodology (supporting); visualization (equal); writing – original draft (supporting); writing – review and editing (supporting). **Tomas Roslin:** Conceptualization (supporting); methodology (supporting); writing – original draft (equal); writing – review and editing (equal).

## BENEFIT‐SHARING STATEMENT

Benefits from this research accrue from the sharing of our data and results in public databases, as described above. All samples for the study were obtained from Finnish beekeepers; the sampling complied with all national rules and legislation; and the results of the research have been shared with the beekeepers.

## Supporting information


Data S1.

## Data Availability

The raw sequence datasets generated during the current study are available in the SRA, in the BioProject PRJNA887226 (https://www.ncbi.nlm.nih.gov/sra/PRJNA887226). The sample metadata is also stored in the SRA, in the same BioProject, using the NCBI package Metagenome or environmental.
