# Supplementary material for: The role of seasonality in shaping the interactions of honeybees with other taxa
Source: Ecol Evol. 2023 Oct 9;13(10):e10580. doi: 10.1002/ece3.10580 (PMC10560870; doi:10.1002/ece3.10580)
Supplement: Supplementary file 1 — Data S1. [file ECE3-13-e10580-s001.docx]

**The role of seasonality in shaping the interactions of honeybees with other taxa**

**Supplemental information**

Helena Wirta, Mirkka Jones, Pablo Peña-Aguilera, Camilo Chacón-Duque, Eero Vesterinen, Otso Ovaskainen, Nerea Abrego, Tomas Roslin

Text S1. *Assessment of three highly and variably abundant taxa*

Among the genera identified based on the sequences, three occur at very high relative read abundances (RRAs) in some samples and have clearly higher RRAs overall than the other genera. They represent three taxonomic groups: the bacterial genus *Apilactobacillus*, the fungal genus *Zygosaccharomyces* and a DNA-virus. The latter was undefined at the genus level, but all the reads assigned to a group labelled Viruses.undefined matched Apis mellifera filamentous virus (AmFV) at the species level. With the genus *Apilactobacillus*, the reads assign to *A. kunkeii*, a lactic acid bacterium living, for example, in the nectar gut of honeybees but commonly detected from flowers as well (Keller et al., 2021; Nowak, Szczuka, Górczyńska, Motyl, & Kręgiel, 2021) and within *Zygosaccharomyces* the reads match *Z. rougii*, a yeast that is able to grow in honey (Kurtzman, 1990; G. Liu et al., 2016). AmFV is a virus commonly detected in honeybees, yet only rarely pathogenic (Hartmann, Forsgren, Charrière, Neumann, & Gauthier, 2015; Quintana et al., 2019). The RRAs of these three genera among the samples reached up to 85.8%, 91.2% and 99.1%, for *Apilactobacillus*, *Zygosaccharomyces* and AmFV, respectively.

The proportions these three highly abundant taxa did not correlate with the total read counts of the samples (Pearson’s correlations *p* = 0.22, 0.73 and 0.78 for *Apilactobacillus*, *Zygosaccharomyces* and AmFV, respectively). The RRAs of *Apilactobacillus* correlated negatively, but weakly, with those of AmFV (Pearson’s correlation -4.462, *p* <0.001) while RRAs of the other predominant taxa did not correlate significantly with each other.

As the relative abundances of these three genera among the DNA reads were very variable, and sometimes very high, they had strong effects on the relative proportions of the other genera found in the samples. Therefore, these three genera and the reads assigned to them were omitted from further analyses based on RRAs.

Moreover, a high abundance of *Zygosaccharomyces* may signal fermentation of particularly moist honey (Kurtzman, 1990; G. Liu et al., 2016). Samples with >80% of all reads assigned to *Zygosaccharomyces* were thus omitted from all further analyses, as we expected strong yeast growth to have compromised other aspects of sample quality.


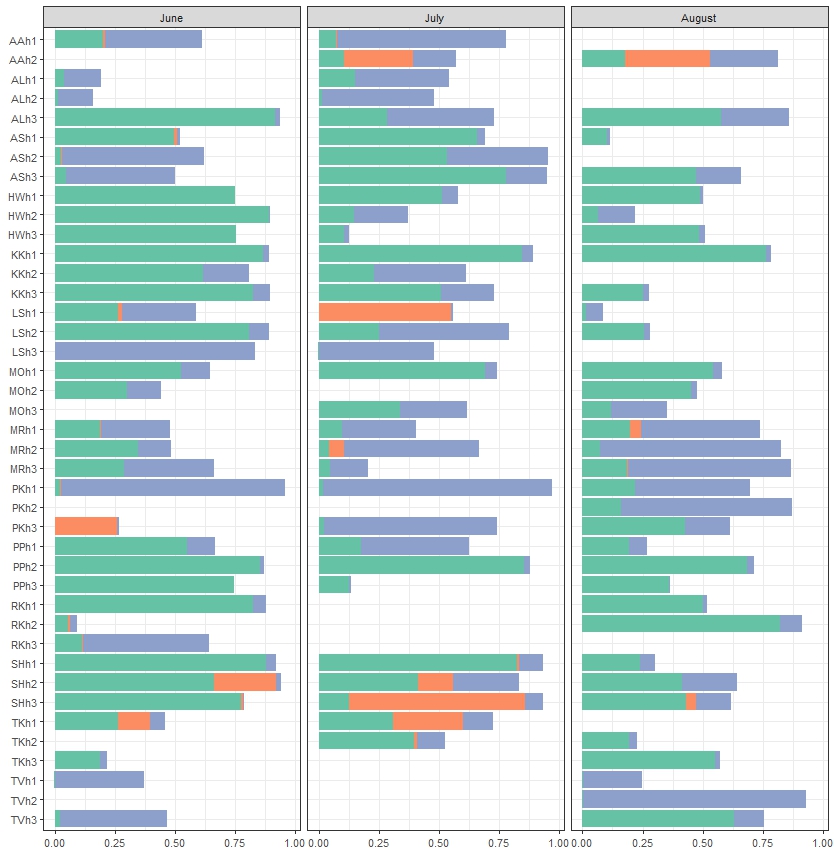


Figure S1. RRAs of three taxa that occurred at extremely high abundances in some honey samples: *Apilactobacillus* (in light green), *Zygosaccharomyces* (orange) and Apis mellifera filamentous virus (AmFV) (light blue), in each hive (rows) per time of summer (columns). The RRAs are calculated based on the total reads mapped per sample.

Text S2. *Relative read abundances of plants and microbes in hives at different times of the season*

The proportions of reads assigned to different kingdoms (plants, bacteria, fungi and viruses) varied considerably between samples (Fig. S2) even after removing the three highly abundant taxa illustrated in Fig. S1 (considering the plant-microbe-RRA, see main text, as used for the abundance model too).

While we include all taxa in the analyses, we illustrate the changes in plants and in microbes (bacteria, fungi and viruses) here separately, as plants and microbes represent different types of encounters and interactions of honeybees. Plants represent food resources while microbes have several functions ranging from being symbiotic gut microbes to pathogens (see main text and table S1). Thus, we calculate plant-RRA based on the reads assigned to plant genera only and microbe-RRA based on reads assigned to bacteria, fungi and viruses (including all genera with mean RRA ≥ 0.01% across samples).


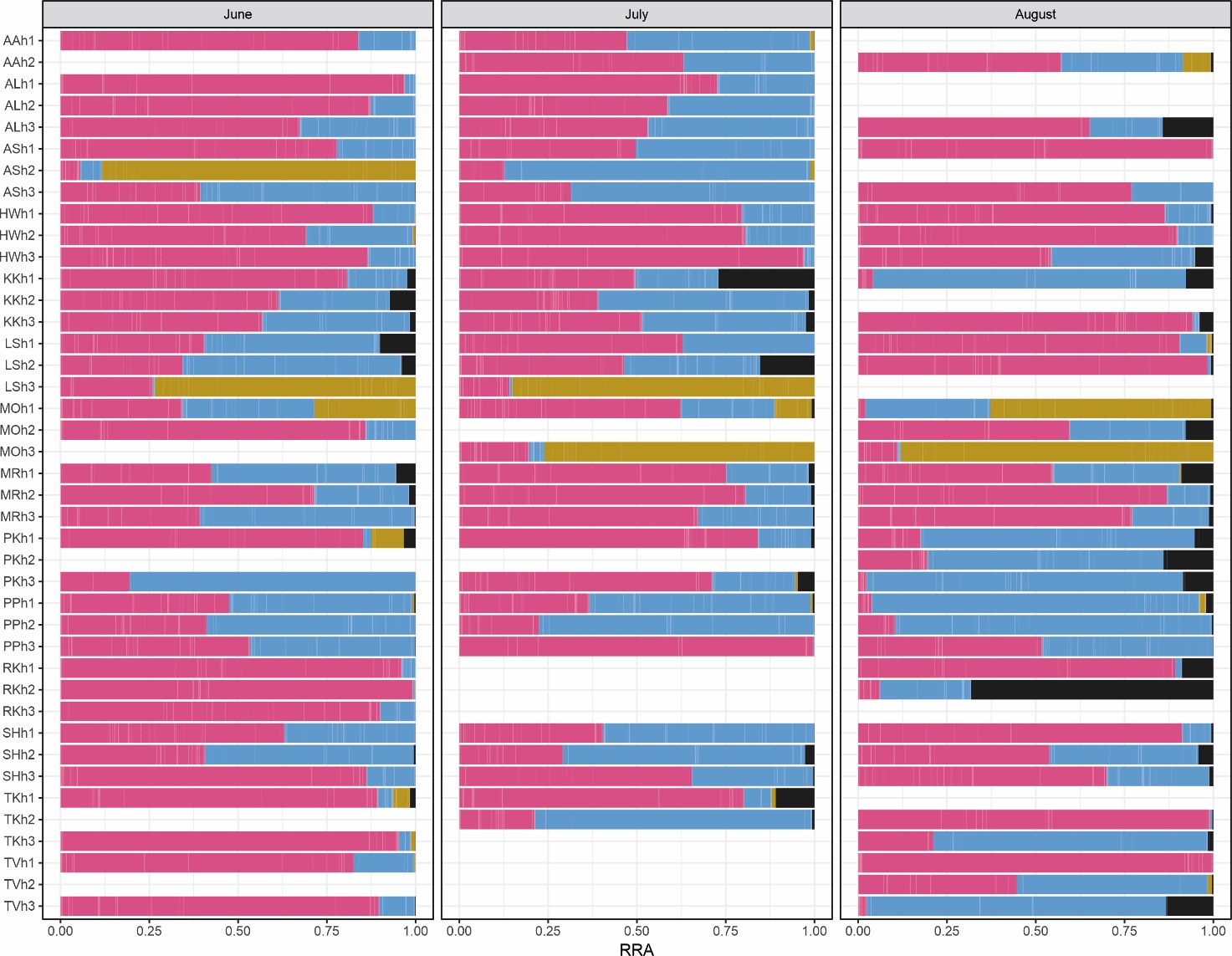


Figure S2. Proportions of taxa belonging to four taxonomic groups detected in each hive (rows) per summer time period (columns), based on plant-microbe-RRA. Plants are shown in pink, bacteria in blue, fungi in orange and viruses in black.

Changes in the relative abundances of plant genera through the summer are slight and gradual in most of the hives (as an example see the the genus-level breakdown of these temporal patterns, e.g. in hives HWh2 and MRh3, illustrated in Fig. S3). Yet, for some hives changes in the plant composition are very clear, especially when *Brassica* begins to dominate in terms of RRA, either in July or August (e.g., in hives ALh1 and ALh3; Fig. S3).

Changes in the RRA of bacterial and fungal genera within hives do not show a clear temporal trajectory over the summer, but in most hives the composition of microbial genera is relatively constant throughout the season. In contrast, there are large differences in microbial composition between hives and hives managed by different beekeepers (Fig. S4). For many hives, a large proportion of the microbial reads come from microbes that commonly occur in honeybee guts, such as *Bombella*, *Parasaccharibacter*, *Gilliamella, Acinetobacter*, *Frischella* and *Acetobacter* (Table S1), with *Frischella* being the most abundant microbe genus in many samples.

Some hives within a single time point had highly contrasting microbial RRAs relative to all others, due to the extreme dominance of some fungal genera, which represented all to nearly all mapped reads (Fig. S4). Examples include hives ASh2, LSh3 and MOh3, where fungal genera displaced bacterial genera in the microbial niche. This pattern was neither beekeeper- nor time-specific, but in these hives the common fungal bee pathogens *Ascoshaera*, causing chalkbrood, and *Aspergillus*, causing stonebrood, had high relative abundances. Both of these fungi require immunocompromised hosts to establish an infection, despite their very high occurrence in hives (Evison & Jensen, 2018; Foley, Fazio, Jensen, & Hughes, 2014a). Thus, based on the high relative proportion of all microbe sequences assigned to these fungal genera, we suspect that these colonies were undergoing an active infection. The genus *Aspergillus*, in turn, has a number of strains that produce antibacterial metabolites, which may reduce bacterial growth (Al-Fakih, Qasem, & Almaqtri, 2019), and hence it is possible that these strains may limit bacteria in either bee hives or in the honeybees themselves. These fungal pathogens are not the only bee pathogens (Table S1) that were encountered in some honey samples at high relative abundances. Another example was *Melissococcus*, which was detected at a high RRA in some hives at certain time points. In some other samples, the fungal genus *Talaromyces* or viruses belonging to the Siphoviridae group were predominant, but the functional role of these taxa is not clear.


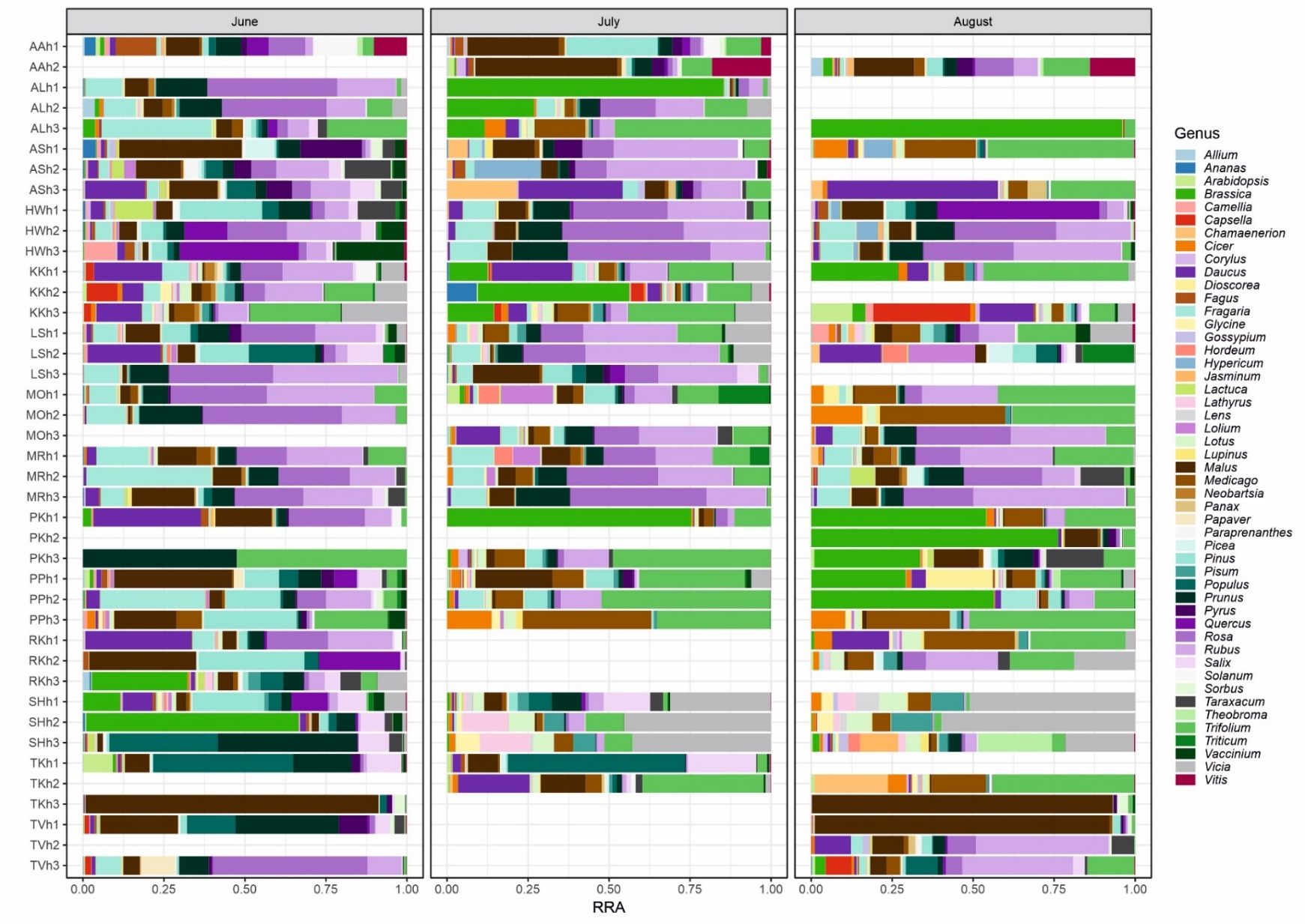


Figure S3. Plant genus RRAs for each hive (in rows) at the three sampled time periods during the summer.


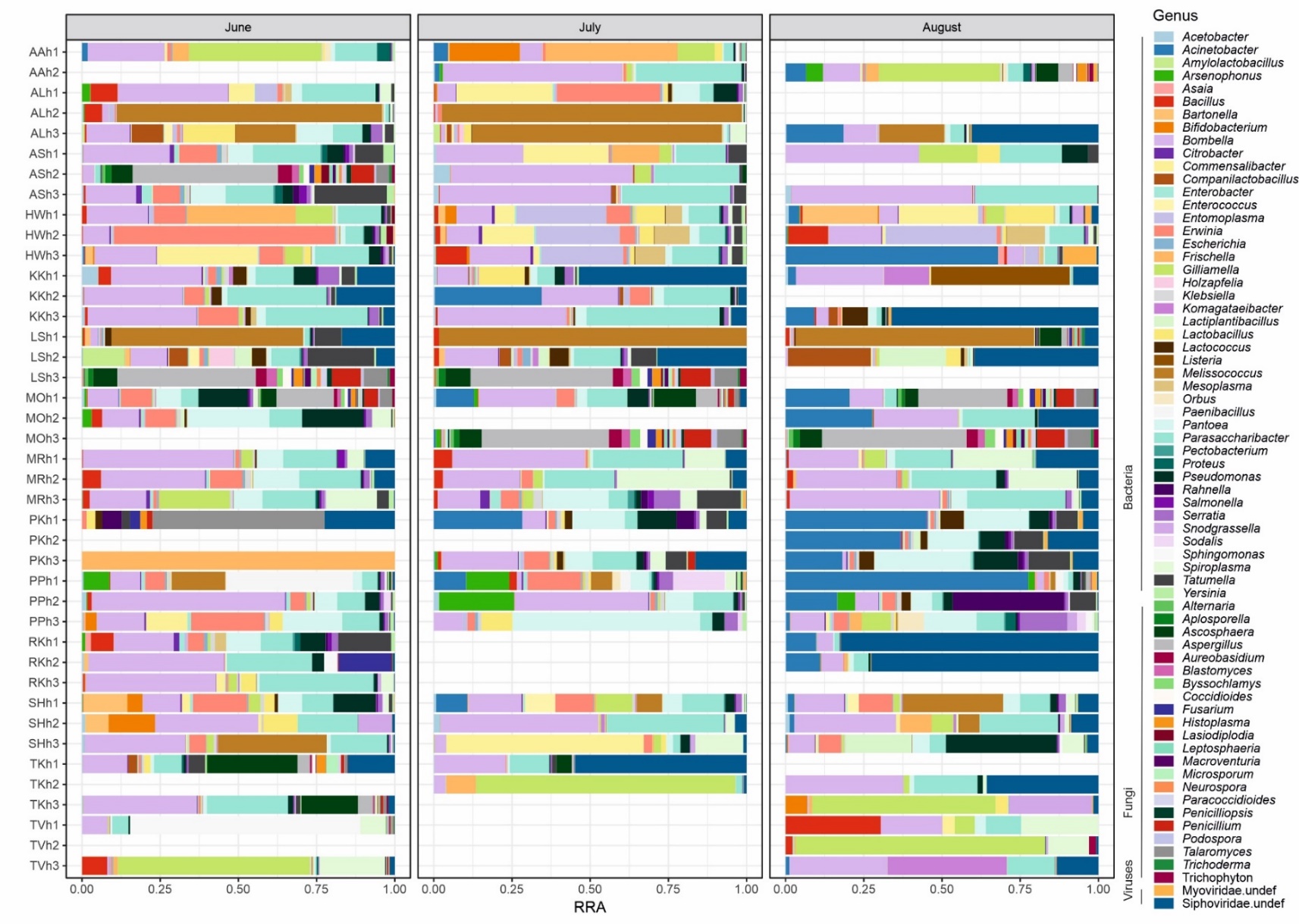


Figure S4. Microbial genus RRAs in each hive (in rows) at the three sampled time periods during the summer.


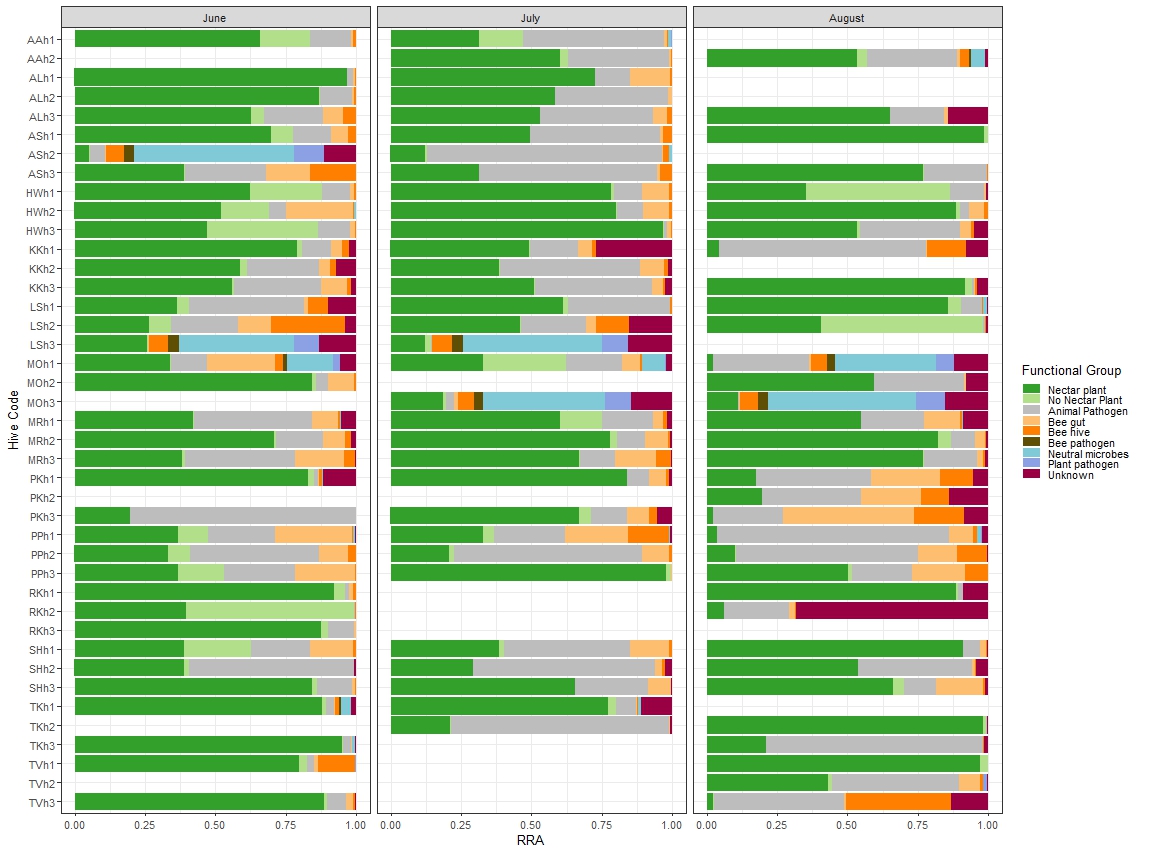


Figure S5. The RRA of different functional groups of plants and microbes, based on plant-microbe-RRA for each hive at the three times of the season. The different functional groups are shown with different colours.


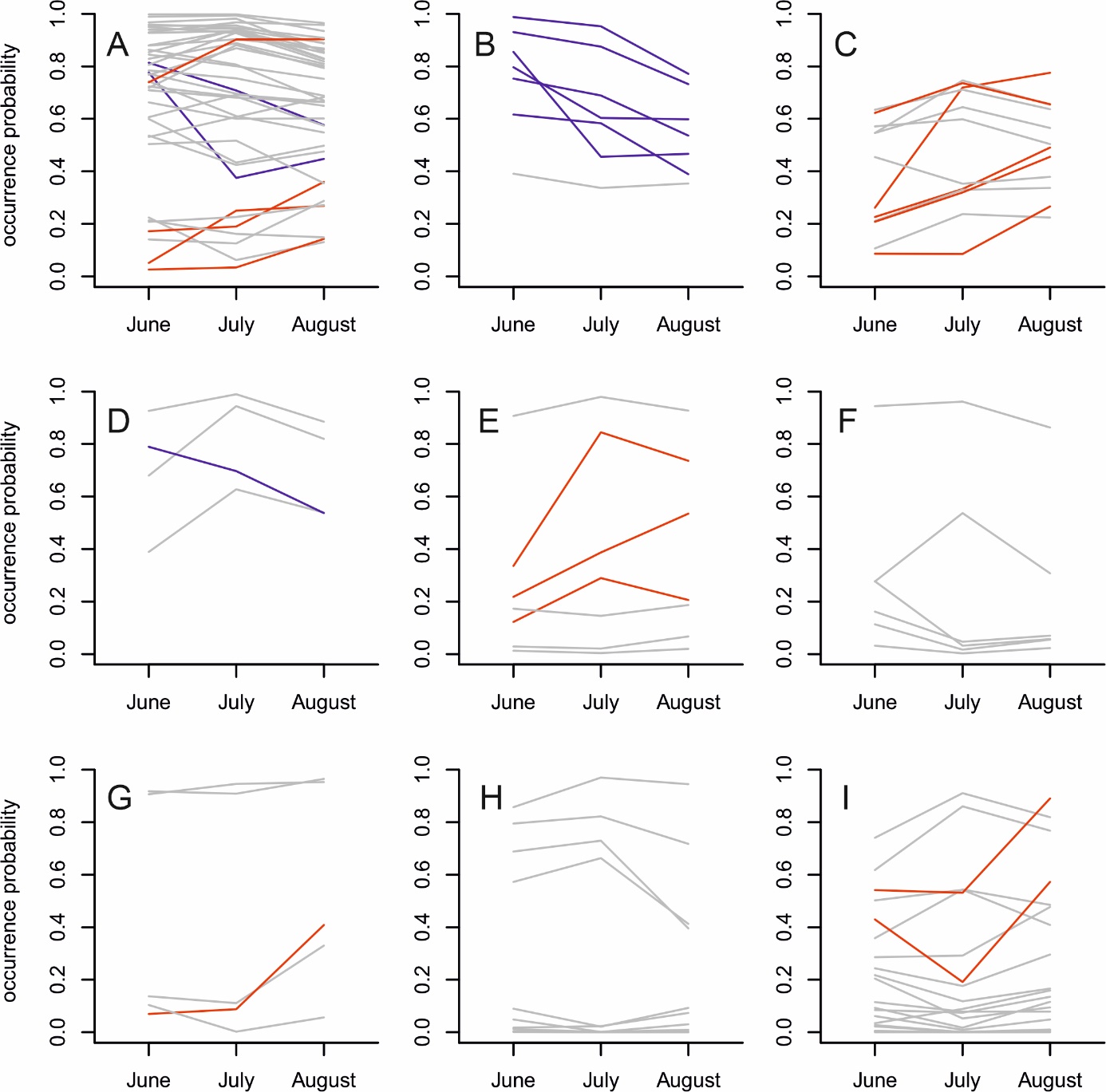


Figure S6. The predicted occurrence probability of each genus at the three times of the season for the different functional groups, as for nectar-producing plants (A), non-nectar producing plants (B), gut microbes (C), hive microbes (D), bee pathogens (E), plant pathogens (F), neutral or positive microbes (G), animal pathogens (H) and unknown (I). Each line shows the posterior mean probability of a genus’s occurrence in June, July and August. Genera for which occurrence probability increases directionally over the season are shown in red (with at least 95% posterior probability for higher occurrence probability in August than in June), whereas genera for which occurrence probability decreases over the season are shown in blue (with at least 95% posterior probability for higher occurrence probability in June than in August).

Text S3. *Does a honey sample collected at the end of the summer compile all the interactions of the summer?*

To assess whether a compound sample collected at the end of the summer reveals a time-integrated estimate of all interactions across the summer, we compared the DNA contents of individual samples from June, July and August to a compound honey sample representing all hives per beekeeper at the end of the summer. For this purpose, we plotted the plant and microbial genera, separately that were detected in the individual monthly samples against the compound sample per beekeeper as Euler diagrams, using the eulerr package in R (Larsson, 2022) and calculated the shared and non-shared taxa.

To illustrate the similarity of the plant and microbe communities of the individual samples from June, July and August, and the compound samples, we use Non-Metric Multidimensional Scaling (NMDS). As the dissimilarity metric, we used the Bray-Curtis dissimilarity index. The function ‘metaMDS’ of the R package vegan (Oksanen et al., 2019), with 1000 iterations and three dimensions was used to illustrate the dissimilarities of the communities among samples.

These compound samples fell short in revealing the diversity of plants, fungi and bacteria exposed by the time-resolved samples (Figs S7 and S8). This finding is in line with expected changes in the taxa encountered by honeybees during the summer, and with the behaviour of the honeybees. If a shortage of nectar resources occurs any time during the summer, the colony will consume honey stored during earlier phases of the summer. As a consequence, such consumption will blur traces of the taxa with which the colony has been in previous contact. Thus, to assess the entire spectrum of taxa that honeybees interact with, we should sample honey at multiple time points during the flowering season.


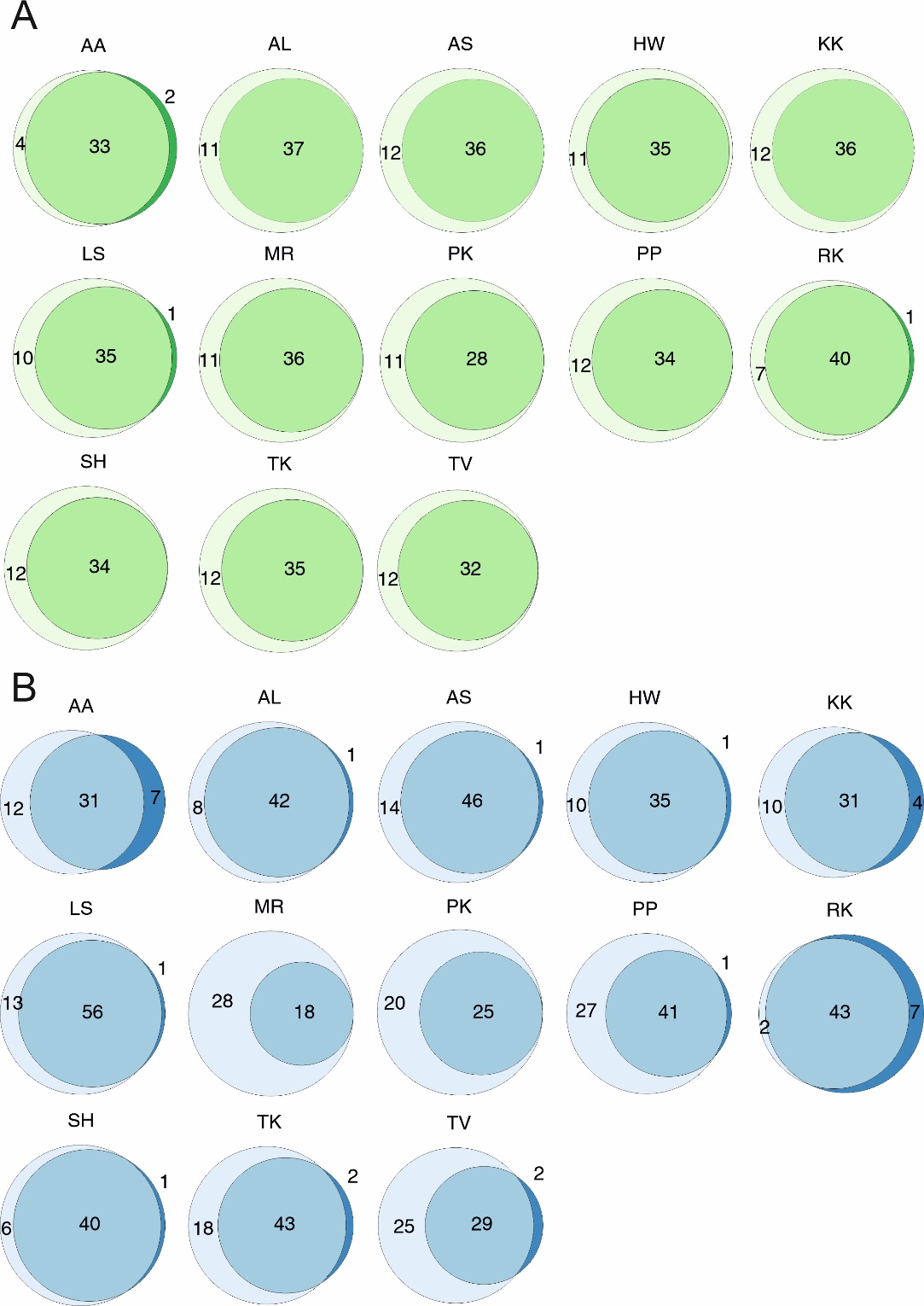


Figure S7. The proportions of shared and non-shared genera, per beekeeper, among the honey samples collected directly from hives at three different time points and the compound honey sample obtained at the end of the summer from all the hives of that beekeeper, for A) plants and B) microbes. The number of genera uniquely detected in the individual samples is given on the left (shown in light green for plants and light blue for microbes), the number of taxa uniquely detected in the compound sample, if any, is given on the right (in dark green for plants and dark blue for microbes) and the genera detected both in the individual and compound samples are shown in the middle of each Euler diagram (in green for plants and in blue for microbes).


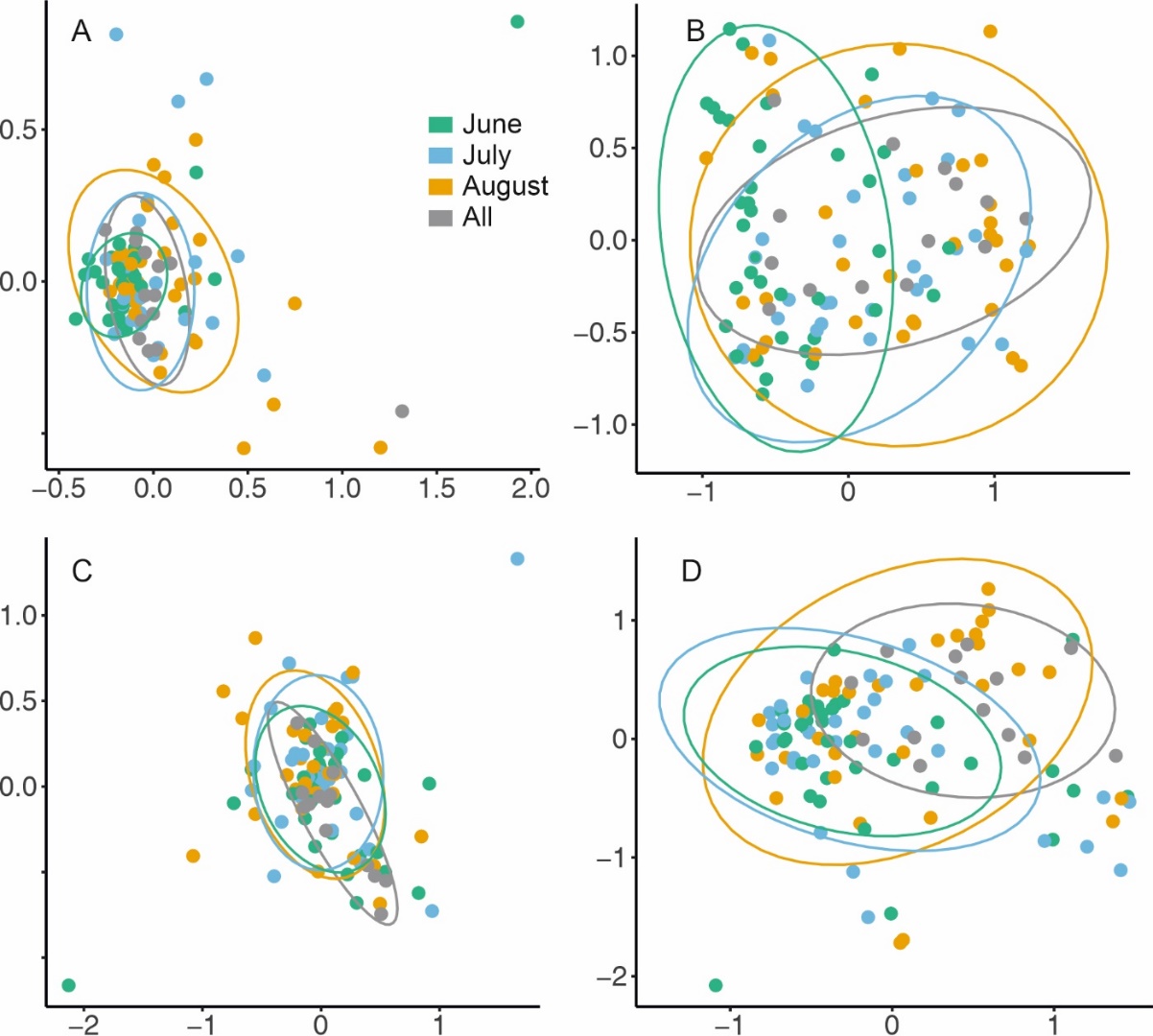


Figure S8. Non-metric multidimensional scaling (NMDS) plots of plant (A and B) and microbe (C and D) communities across the different sampling times and the compound sample “All” at the end of the summer, based on presence-absence data (A and C) and plant-microbe-RRA data (B and D). The ellipses around the samples representing the different time points or the compound sample are drawn including 90% of the samples. The stress values were 0.111, 0.148, 0.124 and 0.163, for ordinations A, B, C and D, respectively.

Most of the taxa detected by the individual samples are detected also by the compound samples, but not all genera were detected either for plants or microbes (Fig. S6). Similarly, while genus-level community composition has considerable overlap whether illustrated based on the samples collected at the separate time points or the compound samples, the community composition of the compound samples is slightly different for that of the individual samples in terms of both plants and microbes (Fig. S7). Thus, using a compound sample at the end of the summer to illustrate and to assess the plant and microbe taxa that honeybees encounter and interact with, does not represent the full diversity of taxa. Many microbial genera, in particular, that were detected within individual samples were not recorded in the final compound samples.

Table S1. Functional group assignments, based on the literature, of honeybee associated genera detected in this study, ordered taxonomically and alphabetically within each taxonomic group. The functional group of taxa considered to be neutral or protective for plants or insects is marked as “neutral or protective”, other groups are written in full.

| Superkingdom | Phylum | Family | Genus | Functional group | Functional based on species | Reference for functional group assignment |
| --- | --- | --- | --- | --- | --- | --- |
| Bacteria | Actinobacteria | Bifidobacteriaceae | *Bifidobacterium* | bee gut microbes | *Bifidobacterium asteroides* | (Raymann & Moran, 2018) |
| Bacteria | Firmicutes | Bacillaceae | *Bacillus* | unknown |  |  |
| Bacteria | Firmicutes | Enterococcaceae | *Enterococcus* | bee gut microbes |  | (Carina Audisio, Torres, Sabaté, Ibarguren, & Apella, 2011) |
| Bacteria | Firmicutes | Enterococcaceae | *Melissococcus* | bee pathogens | *Melissococcus plutonius* | (Fünfhaus, Ebeling, & Genersch, 2018) |
| Bacteria | Firmicutes | Lactobacillaceae | *Amylolactobacillus* | unknown |  |  |
| Bacteria | Firmicutes | Lactobacillaceae | *Apilactobacillus* | bee gut microbes | *Apilactobacillus kunkeei* | (Olofsson & Vásquez, 2008; Raymann & Moran, 2018) |
| Bacteria | Firmicutes | Lactobacillaceae | *Companilactobacillus* | unknown |  |  |
| Bacteria | Firmicutes | Lactobacillaceae | *Holzapfelia* | unknown |  |  |
| Bacteria | Firmicutes | Lactobacillaceae | *Lactiplantibacillus* | bee gut microbes | *Lactiplantibacillus plantarum* | (Iorizzo et al., 2021) |
| Bacteria | Firmicutes | Lactobacillaceae | *Lactobacillus* | bee gut microbes |  | (Olofsson, Alsterfjord, Nilson, Butler, & Vásquez, 2014; Raymann & Moran, 2018) |
| Bacteria | Firmicutes | Listeriaceae | *Listeria* | animal pathogens |  | (Hamon, Bierne, & Cossart, 2006) |
| Bacteria | Firmicutes | Paenibacillaceae | *Paenibacillus* | bee pathogens | *Paenibacillus alvei* | (Fünfhaus et al., 2018) |
| Bacteria | Firmicutes | Streptococcaceae | *Lactococcus* | unknown | *Lactococcus lactis* | (Raymann & Moran, 2018) |
| Bacteria | Proteobacteria | Acetobacteraceae | *Acetobacter* | bee gut microbes |  | (Engel, Martinson, & Moran, 2012) |
| Bacteria | Proteobacteria | Acetobacteraceae | *Asaia* | unknown |  |  |
| Bacteria | Proteobacteria | Acetobacteraceae | *Bombella* | bee gut microbes | *Bombella apis* | (Smith, Anderson, Corby-Harris, McFrederick, & Newton, 2020) |
| Bacteria | Proteobacteria | Acetobacteraceae | *Commensalibacter* | bee gut microbes |  | (Dong et al., 2020) |
| Bacteria | Proteobacteria | Acetobacteraceae | *Komagataeibacter* | unknown |  |  |
| Bacteria | Proteobacteria | Acetobacteraceae | *Parasaccharibacter* | bee hive microbes | *Parasaccharibacter apium* | (Corby-Harris et al., 2016, 2014) |
| Bacteria | Proteobacteria | Bartonellaceae | *Bartonella* | bee gut microbes | *Bartonella apis* | (Kešnerová, Moritz, & Engel, 2016; Raymann & Moran, 2018) |
| Bacteria | Proteobacteria | Bruguierivoracaceae | *Sodalis* | unknown |  |  |
| Bacteria | Proteobacteria | Enterobacteriaceae | *Citrobacter* | animal pathogens | *Citrobacter freundii* | (Erban et al., 2017; L. H. Liu et al., 2018) |
| Bacteria | Proteobacteria | Enterobacteriaceae | *Enterobacter* | bee gut microbes |  | (Piva et al., 2020) |
| Bacteria | Proteobacteria | Enterobacteriaceae | *Escherichia* | animal pathogens | *Escherichia coli* | (Allocati, Masulli, Alexeyev, & Ilio, 2013; Kaper, Nataro, & Mobley, 2004) |
| Bacteria | Proteobacteria | Enterobacteriaceae | *Klebsiella* | bee hive microbes | *Klebsiella pneumoniae* | (Khan, Somerville, Frese, & Nayudu, 2020; Raymann & Moran, 2018) |
| Bacteria | Proteobacteria | Enterobacteriaceae | *Salmonella* | animal pathogens | *Salmonella enterica* | (Knodler & Elfenbein, 2019) |
| Bacteria | Proteobacteria | Erwiniaceae | *Erwinia* | plant pathogens | *Erwinia tasmaniensis* | (Huang, Phillippe, & Phillippe, 1990; Johnson, Stockwell, Burgett, Sugar, & Loper, 1993) |
| Bacteria | Proteobacteria | Erwiniaceae | *Pantoea* | neutral or protective | *Pantoea agglomerans* | (Loncaric et al., 2009) |
| Bacteria | Proteobacteria | Erwiniaceae | *Tatumella* | unknown |  |  |
| Bacteria | Proteobacteria | Moraxellaceae | *Acinetobacter* | bee gut microbes | *Acinetobacter apium* | (Kim et al., 2014) |
| Bacteria | Proteobacteria | Morganellaceae | *Arsenophonus* | bee pathogens | *Arsenophonus nasoniae* | (Budge et al., 2016; Yañez, Gauthier, Chantawannakul, & Neumann, 2016) |
| Bacteria | Proteobacteria | Morganellaceae | *Proteus* | unknown |  |  |
| Bacteria | Proteobacteria | Neisseriaceae | *Snodgrassella* | bee gut microbes | *Snodgrassella alvi* | (Raymann & Moran, 2018) |
| Bacteria | Proteobacteria | Orbaceae | *Frischella* | bee gut microbes | *Frischella perrara* | (Raymann & Moran, 2018) |
| Bacteria | Proteobacteria | Orbaceae | *Gilliamella* | bee gut microbes | *Gilliamella apicola* | (Raymann & Moran, 2018) |
| Bacteria | Proteobacteria | Orbaceae | *Orbus* | unknown |  |  |
| Bacteria | Proteobacteria | Pectobacteriaceae | *Pectobacterium* | plant pathogens |  | (Czajkowski, Pé, Van Veen, & Van Der Wolf, n.d.) |
| Bacteria | Proteobacteria | Pseudomonadaceae | *Pseudomonas* | neutral or protective | *Pseudomonas syringae* | (Johnson et al., 1993) |
| Bacteria | Proteobacteria | Sphingomonadaceae | *Sphingomonas* | unknown |  |  |
| Bacteria | Proteobacteria | Yersiniaceae | *Rahnella* | bee hive microbes | *Rahnella aquatilis* | (Hubert et al., 2015; Kačániová, Gasper, & Terentjeva, 2019) |
| Bacteria | Proteobacteria | Yersiniaceae | *Serratia* | bee pathogens |  | (Fünfhaus et al., 2018) |
| Bacteria | Proteobacteria | Yersiniaceae | *Yersinia* | animal pathogens | *Yersinia ruckeri* | (Achtman et al., 1999; Kumar, Menanteau-Ledouble, Saleh, & El-Matbouli, 2015) |
| Bacteria | Tenericutes | Entomoplasmataceae | *Entomoplasma* | neutral or protective | *Entomoplasma melaleucae* | (Gasparich, 2014) |
| Bacteria | Tenericutes | Entomoplasmataceae | *Mesoplasma* | neutral or protective | *Mesoplasma florum* | (Baby et al., 2018; Gasparich, 2014; McCoy, Basham, & Tully, 1984) |
| Bacteria | Tenericutes | Spiroplasmataceae | *Spiroplasma* | bee pathogens | *Spiroplasma melliferum* | (Fünfhaus et al., 2018) |
| Eukaryota | Ascomycota | Ajellomycetaceae | *Blastomyces* | animal pathogens | *Blastomyces gilchristii* | (Bagal et al., 2022; Dalcin, Rothstein, Spinato, Escott, & Kus, 2016) |
| Eukaryota | Ascomycota | Ajellomycetaceae | *Histoplasma* | animal pathogens | *Histoplasma capsulatum* | (Mittal, Ponce, Gendlina, & Nosanchuk, 2019; Wheat, Byron, Batteiger, & Boonmee Sathapatayavongs, 1990) |
| Eukaryota | Ascomycota | Aplosporellaceae | *Aplosporella* | unknown |  |  |
| Eukaryota | Ascomycota | Arthrodermataceae | *Microsporum* | animal pathogens | *Microsporum canis* | (Aneke, Otranto, & Cafarchia, 2018) |
| Eukaryota | Ascomycota | Arthrodermataceae | *Trichophyton* | animal pathogens | *Trichophyton verrucosum* | (Papini, Nardoni, Fanelli, & Mancianti, 2009) |
| Eukaryota | Ascomycota | Ascosphaeraceae | *Ascosphaera* | bee pathogens | *Ascosphaera apis* | (Evison & Jensen, 2018) |
| Eukaryota | Ascomycota | Aspergillaceae | *Aspergillus* | bee pathogens | *Aspergillus niger* | (Foley, Fazio, Jensen, & Hughes, 2014b; Shoreit & Bagy, 1995) |
| Eukaryota | Ascomycota | Aspergillaceae | *Penicilliopsis* | unknown |  |  |
| Eukaryota | Ascomycota | Aspergillaceae | *Penicillium* | unknown |  |  |
| Eukaryota | Ascomycota | Botryosphaeriaceae | *Lasiodiplodia* | plant pathogens | *Lasiodiplodia theobromae* | (Salvatore, Andolfi, & Nicoletti, 2020) |
| Eukaryota | Ascomycota | Chaetomiaceae | *Podospora* | unknown |  |  |
| Eukaryota | Ascomycota | Didymellaceae | *Macroventuria* | unknown |  |  |
| Eukaryota | Ascomycota | Hypocreaceae | *Trichoderma* | neutral or protective |  | (Contreras-Cornejo, Macías-Rodríguez, Cortés-Penagos, & Ló Pez-Bucio, n.d.) |
| Eukaryota | Ascomycota | Leptosphaeriaceae | *Leptosphaeria* | plant pathogens | *Leptosphaeria maculans* | (Kutcher, Keri, McLaren, & Rimmer, 2007) |
| Eukaryota | Ascomycota | Nectriaceae | *Fusarium* | plant pathogens | *Fusarium vanettenii* | (Debbarma, Kamil, Maya Bashyal, Choudhary, & Thokla, 2021; Ma et al., 2013) |
| Eukaryota | Ascomycota | Onygenales-undef | *Coccidioides* | animal pathogens | *Coccidioides posadasii* | (Kollath, Miller, & Barker, 2019) |
| Eukaryota | Ascomycota | Onygenales-undef | *Paracoccidioides* | animal pathogens | *Paracoccidioides brasiliensis* | (Borges-Walmsley, Chen, Shu, & Walmsley, 2002) |
| Eukaryota | Ascomycota | Pleosporaceae | *Alternaria* | plant pathogens | *Alternaria alternata* | (Tsuge et al., 2013) |
| Eukaryota | Ascomycota | Saccharomycetaceae | *Zygosaccharomyces* | bee hive microbes | *Zygosaccharomyces rouxii* | (Kurtzman, 1990) |
| Eukaryota | Ascomycota | Saccotheciaceae | *Aureobasidium* | unknown |  |  |
| Eukaryota | Ascomycota | Sordariaceae | *Neurospora* | unknown |  |  |
| Eukaryota | Ascomycota | Thermoascaceae | *Byssochlamys* | unknown |  |  |
| Eukaryota | Ascomycota | Trichocomaceae | *Talaromyces* | unknown |  |  |
| Eukaryota | Streptophyta | Amaryllidaceae | *Allium* | nectar producing plants |  |  |
| Eukaryota | Streptophyta | Apiaceae | *Daucus* | nectar producing plants |  |  |
| Eukaryota | Streptophyta | Araliaceae | *Panax* | nectar producing plants |  |  |
| Eukaryota | Streptophyta | Asteraceae | *Lactuca* | nectar producing plants |  |  |
| Eukaryota | Streptophyta | Asteraceae | *Paraprenanthes* | nectar producing plants |  |  |
| Eukaryota | Streptophyta | Asteraceae | *Taraxacum* | nectar producing plants |  |  |
| Eukaryota | Streptophyta | Betulaceae | *Corylus* | nectar producing plants |  |  |
| Eukaryota | Streptophyta | Brassicaceae | *Arabidopsis* | nectar producing plants |  |  |
| Eukaryota | Streptophyta | Brassicaceae | *Brassica* | nectar producing plants |  |  |
| Eukaryota | Streptophyta | Brassicaceae | *Capsella* | nectar producing plants |  |  |
| Eukaryota | Streptophyta | Bromeliaceae | *Ananas* | nectar producing plants |  |  |
| Eukaryota | Streptophyta | Dioscoreaceae | *Dioscorea* | nectar producing plants |  |  |
| Eukaryota | Streptophyta | Ericaceae | *Vaccinium* | nectar producing plants |  |  |
| Eukaryota | Streptophyta | Fabaceae | *Cicer* | nectar producing plants |  |  |
| Eukaryota | Streptophyta | Fabaceae | *Glycine* | nectar producing plants |  |  |
| Eukaryota | Streptophyta | Fabaceae | *Lathyrus* | nectar producing plants |  |  |
| Eukaryota | Streptophyta | Fabaceae | *Lens* | nectar producing plants |  |  |
| Eukaryota | Streptophyta | Fabaceae | *Lotus* | nectar producing plants |  |  |
| Eukaryota | Streptophyta | Fabaceae | *Lupinus* | nectar producing plants |  |  |
| Eukaryota | Streptophyta | Fabaceae | *Medicago* | nectar producing plants |  |  |
| Eukaryota | Streptophyta | Fabaceae | *Pisum* | nectar producing plants |  |  |
| Eukaryota | Streptophyta | Fabaceae | *Trifolium* | nectar producing plants |  |  |
| Eukaryota | Streptophyta | Fabaceae | *Vicia* | nectar producing plants |  |  |
| Eukaryota | Streptophyta | Fagaceae | *Fagus* | plants not producing nectar |  |  |
| Eukaryota | Streptophyta | Fagaceae | *Quercus* | plants not producing nectar |  |  |
| Eukaryota | Streptophyta | Hypericaceae | *Hypericum* | nectar producing plants |  |  |
| Eukaryota | Streptophyta | Malvaceae | *Gossypium* | nectar producing plants |  |  |
| Eukaryota | Streptophyta | Malvaceae | *Theobroma* | nectar producing plants |  |  |
| Eukaryota | Streptophyta | Oleaceae | *Jasminum* | nectar producing plants |  |  |
| Eukaryota | Streptophyta | Onagraceae | *Chamaenerion* | nectar producing plants |  |  |
| Eukaryota | Streptophyta | Orobanchaceae | *Neobartsia* | nectar producing plants |  |  |
| Eukaryota | Streptophyta | Papaveraceae | *Papaver* | nectar producing plants |  |  |
| Eukaryota | Streptophyta | Pinaceae | *Picea* | plants not producing nectar |  |  |
| Eukaryota | Streptophyta | Pinaceae | *Pinus* | plants not producing nectar |  |  |
| Eukaryota | Streptophyta | Poaceae | *Hordeum* | plants not producing nectar |  |  |
| Eukaryota | Streptophyta | Poaceae | *Lolium* | plants not producing nectar |  |  |
| Eukaryota | Streptophyta | Poaceae | *Triticum* | plants not producing nectar |  |  |
| Eukaryota | Streptophyta | Rosaceae | *Fragaria* | nectar producing plants |  |  |
| Eukaryota | Streptophyta | Rosaceae | *Malus* | nectar producing plants |  |  |
| Eukaryota | Streptophyta | Rosaceae | *Prunus* | nectar producing plants |  |  |
| Eukaryota | Streptophyta | Rosaceae | *Pyrus* | nectar producing plants |  |  |
| Eukaryota | Streptophyta | Rosaceae | *Rosa* | nectar producing plants |  |  |
| Eukaryota | Streptophyta | Rosaceae | *Rubus* | nectar producing plants |  |  |
| Eukaryota | Streptophyta | Rosaceae | *Sorbus* | nectar producing plants |  |  |
| Eukaryota | Streptophyta | Salicaceae | *Populus* | nectar producing plants | *Populus trichocarpa* | (Escalante-Pérez et al., 2012) |
| Eukaryota | Streptophyta | Salicaceae | *Salix* | nectar producing plants |  | (Ostaff et al., 2015) |
| Eukaryota | Streptophyta | Solanaceae | *Solanum* | nectar producing plants |  |  |
| Eukaryota | Streptophyta | Theaceae | *Camellia* | nectar producing plants |  |  |
| Eukaryota | Streptophyta | Vitaceae | *Vitis* | nectar producing plants |  |  |
| Viruses | Viruses | Myoviridae | *Myoviridae.undef* | unknown |  |  |
| Viruses | Viruses | na | *Viruses.undef* | bee pathogens | *Apis mellifera filamentous virus* | (Hartmann et al., 2015) |
| Viruses | Viruses | Siphoviridae | *Siphoviridae.undef* | unknown |  |  |

Table S2. Order of taxa and the variance partitioning for each taxa for the presence-absence model as well as the Tjur R^2^ value as a measure of variance explained. The order of taxa is as shown in figures. Given are all the taxa that were available for the model, but the order number and the variance partitioning are given only to taxa that were included.

| Taxonomic group |  |  | fixed | total reads | random |  |  |  |  |
| --- | --- | --- | --- | --- | --- | --- | --- | --- | --- |
|  | Genus | order | time |  | sample | site | hive | beekeeper | Tjur R^2^ |
| plants | *Allium* | 1 | 1.7 | 0.7 | 11.8 | 1.1 | 3.2 | 1.2 | 19.7 |
|  | *Ananas* | 2 | 6.0 | 3.7 | 5.6 | 4.6 | 2.1 | 1.6 | 23.6 |
|  | *Arabidopsis* | 3 | 1.6 | 0.9 | 20.1 | 2.1 | 4.6 | 0.6 | 30.0 |
|  | *Brassica* | 4 | 2.4 | 1.3 | 13.7 | 1.2 | 2.7 | 1.2 | 22.4 |
|  | *Camellia* | 5 | 5.7 | 2.1 | 22.8 | 5.9 | 2.7 | 0.6 | 39.7 |
|  | *Capsella* | 6 | 2.1 | 1.1 | 6.4 | 5.8 | 12.9 | 12.5 | 40.8 |
|  | *Chamaenerion* | 7 | 11.1 | 4.3 | 2.5 | 5.5 | 2.2 | 2.0 | 27.6 |
|  | *Cicer* | 8 | 3.0 | 1.0 | 5.3 | 0.9 | 2.1 | 0.9 | 13.1 |
|  | *Corylus* | 9 | 2.5 | 1.2 | 27.2 | 17.2 | 3.6 | 0.6 | 52.2 |
|  | *Daucus* | 10 | 3.7 | 3.9 | 11.8 | 1.8 | 1.6 | 2.9 | 25.8 |
|  | *Dioscorea* | 11 | 8.8 | 2.9 | 3.5 | 1.2 | 4.4 | 0.6 | 21.4 |
|  | *Fagus* | 12 | 5.8 | 2.2 | 31.6 | 8.8 | 2.8 | 0.5 | 51.6 |
|  | *Fragaria* | 13 | 3.6 | 3.2 | 5.6 | 1.1 | 1.1 | 1.2 | 15.9 |
|  | *Glycine* | 14 | 2.1 | 0.8 | 11.8 | 2.2 | 3.0 | 5.7 | 25.6 |
|  | *Gossypium* | 15 | 1.6 | 1.0 | 10.4 | 17.3 | 5.0 | 1.9 | 37.2 |
|  | *Hordeum* | 16 | 2.7 | 2.2 | 8.8 | 7.9 | 4.0 | 1.9 | 27.6 |
|  | *Hypericum* | 17 | 6.6 | 2.1 | 3.1 | 9.4 | 2.1 | 0.9 | 24.2 |
|  | *Jasminum* | 18 | 1.9 | 2.3 | 24.1 | 8.4 | 2.8 | 0.8 | 40.4 |
|  | *Lactuca* | 19 | 0.7 | 0.4 | 60.9 | 0.7 | 0.5 | 0.5 | 63.7 |
|  | *Lathyrus* | 20 | 1.9 | 0.6 | 1.9 | 65.2 | 0.4 | 0.5 | 70.6 |
|  | *Lens* | 21 | 1.7 | 1.0 | 2.1 | 23.1 | 3.2 | 0.6 | 31.7 |
|  | *Lolium* | 22 | 10.0 | 5.3 | 10.6 | 4.0 | 4.4 | 2.7 | 37.0 |
|  | *Lotus* | 23 | 2.9 | 1.2 | 5.1 | 1.9 | 7.6 | 2.6 | 21.4 |
|  | *Lupinus* | 24 | 3.5 | 2.7 | 9.1 | 3.3 | 2.1 | 1.2 | 21.9 |
|  | *Malus* |  |  |  |  |  |  |  |  |
|  | *Medicago* | 25 | 4.8 | 1.6 | 8.5 | 1.3 | 1.0 | 0.8 | 18.1 |
|  | *Neobartsia* | 26 | 0.8 | 0.5 | 9.2 | 1.9 | 0.9 | 45.4 | 58.6 |
|  | *Panax* | 27 | 1.5 | 0.9 | 15.3 | 5.0 | 1.1 | 1.1 | 24.9 |
|  | *Papaver* | 28 | 1.4 | 0.7 | 14.0 | 2.9 | 1.2 | 2.7 | 22.9 |
|  | *Paraprenanthes* | 29 | 1.2 | 1.1 | 61.2 | 0.3 | 0.3 | 0.5 | 64.7 |
|  | *Picea* | 30 | 8.7 | 3.8 | 5.8 | 3.2 | 2.8 | 0.9 | 25.1 |
|  | *Pinus* | 31 | 9.5 | 2.6 | 23.0 | 2.4 | 5.4 | 1.1 | 44.0 |
|  | *Pisum* | 32 | 3.1 | 1.1 | 4.1 | 6.5 | 3.7 | 0.8 | 19.4 |
|  | *Populus* | 33 | 6.8 | 3.3 | 20.3 | 1.7 | 1.0 | 1.6 | 34.6 |
|  | *Prunus* |  |  |  |  |  |  |  |  |
|  | *Pyrus* | 34 | 2.8 | 1.7 | 26.0 | 8.2 | 3.6 | 0.9 | 43.3 |
|  | *Quercus* | 35 | 7.1 | 9.5 | 11.3 | 9.0 | 4.5 | 0.7 | 42.0 |
|  | *Rosa* |  |  |  |  |  |  |  |  |
|  | *Rubus* | 36 | 5.9 | 3.8 | 8.7 | 4.0 | 2.3 | 0.7 | 25.4 |
|  | *Salix* | 37 | 4.6 | 4.8 | 36.5 | 0.9 | 1.6 | 0.7 | 49.1 |
|  | *Solanum* | 38 | 1.7 | 0.7 | 13.1 | 12.3 | 3.3 | 1.9 | 33.0 |
|  | *Sorbus* | 39 | 1.6 | 2.1 | 15.4 | 0.9 | 0.9 | 4.1 | 25.0 |
|  | *Taraxacum* | 40 | 1.0 | 0.4 | 58.0 | 0.9 | 0.3 | 0.3 | 60.9 |
|  | *Theobroma* | 41 | 1.5 | 0.7 | 19.1 | 6.5 | 4.3 | 1.8 | 33.9 |
|  | *Trifolium* | 42 | 5.5 | 2.2 | 2.4 | 1.7 | 1.7 | 1.3 | 14.8 |
|  | *Triticum* | 43 | 7.3 | 2.3 | 8.3 | 12.1 | 8.9 | 6.1 | 45.0 |
|  | *Vaccinium* | 44 | 4.4 | 3.1 | 14.1 | 9.7 | 2.0 | 0.8 | 34.2 |
|  | *Vicia* | 45 | 1.6 | 1.0 | 4.0 | 49.2 | 1.7 | 0.7 | 58.3 |
|  | *Vitis* | 46 | 2.8 | 2.3 | 30.8 | 17.1 | 4.2 | 0.6 | 57.8 |
| bacteria | *Acetobacter* | 47 | 2.7 | 1.5 | 6.7 | 0.8 | 1.3 | 1.7 | 14.7 |
|  | *Acinetobacter* | 48 | 6.8 | 1.0 | 20.2 | 1.0 | 1.4 | 1.3 | 31.8 |
|  | *Amylolactobacillus* |  |  |  |  |  |  |  |  |
|  | *Apilactobacillus* |  |  |  |  |  |  |  |  |
|  | *Arsenophonus* | 49 | 4.5 | 3.4 | 16.4 | 2.2 | 5.2 | 1.0 | 32.8 |
|  | *Asaia* | 50 | 1.8 | 1.8 | 2.1 | 0.9 | 2.9 | 0.5 | 10.0 |
|  | *Bacillus* | 51 | 2.9 | 5.4 | 11.6 | 1.7 | 1.8 | 10.6 | 33.9 |
|  | *Bartonella* | 52 | 1.7 | 2.8 | 3.5 | 0.5 | 0.7 | 0.8 | 10.0 |
|  | *Bifidobacterium* | 53 | 2.5 | 4.7 | 5.3 | 2.9 | 6.6 | 1.0 | 23.1 |
|  | *Bombella* |  |  |  |  |  |  |  |  |
|  | *Citrobacter* | 54 | 2.4 | 12.9 | 54.5 | 1.2 | 0.6 | 0.9 | 72.5 |
|  | *Commensalibacter* | 55 | 4.7 | 2.3 | 9.6 | 1.5 | 6.4 | 0.8 | 25.3 |
|  | *Companilactobacillus* | 56 | 1.4 | 1.0 | 1.0 | 0.6 | 2.0 | 0.6 | 6.6 |
|  | *Enterobacter* | 57 | 1.9 | 10.4 | 46.8 | 1.7 | 0.6 | 0.8 | 62.2 |
|  | *Enterococcus* | 58 | 3.2 | 0.9 | 13.8 | 1.9 | 2.6 | 0.5 | 23.0 |
|  | *Entomoplasma* | 59 | 1.1 | 0.5 | 10.4 | 1.0 | 2.2 | 52.3 | 67.4 |
|  | *Erwinia* | 60 | 6.1 | 7.2 | 27.7 | 0.9 | 1.0 | 0.8 | 43.8 |
|  | *Escherichia* | 61 | 2.7 | 6.6 | 66.4 | 0.6 | 0.3 | 0.5 | 77.1 |
|  | *Frischella* | 62 | 1.4 | 2.9 | 5.7 | 1.1 | 9.2 | 2.8 | 23.1 |
|  | *Gilliamella* | 63 | 2.4 | 1.4 | 8.2 | 1.4 | 6.9 | 6.8 | 27.1 |
|  | *Holzapfelia* |  |  |  |  |  |  |  |  |
|  | *Klebsiella* | 64 | 5.1 | 5.4 | 55.4 | 0.4 | 0.3 | 0.4 | 67.0 |
|  | *Komagataeibacter* | 65 | 1.8 | 6.9 | 7.4 | 0.7 | 1.2 | 0.8 | 18.7 |
|  | *Lactiplantibacillus* | 66 | 5.8 | 0.9 | 2.4 | 3.3 | 1.5 | 1.3 | 15.3 |
|  | *Lactobacillus* | 67 | 4.3 | 0.9 | 12.5 | 1.1 | 3.9 | 1.3 | 24.0 |
|  | *Lactococcus* | 68 | 2.8 | 1.5 | 14.5 | 2.0 | 1.5 | 8.1 | 30.4 |
|  | *Listeria* | 69 | 2.9 | 1.4 | 4.6 | 1.7 | 1.0 | 1.1 | 12.7 |
|  | *Melissococcus* | 70 | 3.7 | 4.8 | 2.0 | 15.6 | 3.1 | 1.8 | 31.0 |
|  | *Mesoplasma* | 71 | 2.4 | 0.6 | 13.2 | 0.8 | 1.2 | 50.7 | 68.8 |
|  | *Orbus* | 72 | 2.5 | 6.5 | 5.1 | 1.5 | 5.3 | 6.9 | 27.8 |
|  | *Paenibacillus* | 73 | 2.1 | 3.6 | 5.0 | 3.4 | 0.9 | 0.8 | 15.8 |
|  | *Pantoea* | 74 | 3.4 | 2.9 | 26.2 | 1.0 | 1.9 | 0.8 | 36.3 |
|  | *Parasaccharibacter* | 75 | 5.3 | 4.9 | 6.7 | 1.4 | 2.6 | 1.3 | 22.3 |
|  | *Pectobacterium* | 76 | 2.2 | 4.9 | 56.0 | 0.8 | 0.6 | 0.5 | 65.0 |
|  | *Proteus* | 77 | 1.3 | 7.6 | 33.1 | 0.5 | 0.6 | 1.4 | 44.5 |
|  | *Pseudomonas* | 78 | 2.8 | 6.8 | 21.7 | 1.3 | 5.9 | 1.0 | 39.4 |
|  | *Rahnella* | 79 | 1.8 | 0.8 | 44.4 | 0.7 | 1.0 | 1.8 | 50.5 |
|  | *Salmonella* | 80 | 4.3 | 9.0 | 50.7 | 0.7 | 1.5 | 1.0 | 67.3 |
|  | *Serratia* | 81 | 3.4 | 6.7 | 52.0 | 0.5 | 0.4 | 0.7 | 63.6 |
|  | *Snodgrassella* | 82 | 2.2 | 3.2 | 3.9 | 1.0 | 5.2 | 8.1 | 23.7 |
|  | *Sodalis* | 83 | 1.4 | 2.2 | 34.4 | 0.6 | 0.9 | 0.8 | 40.3 |
|  | *Sphingomonas* | 84 | 1.5 | 11.7 | 3.9 | 1.5 | 2.4 | 6.4 | 27.3 |
|  | *Spiroplasma* | 85 | 7.8 | 2.7 | 6.4 | 5.1 | 1.6 | 15.0 | 38.5 |
|  | *Tatumella* | 86 | 2.1 | 4.4 | 25.0 | 1.3 | 3.1 | 1.8 | 37.8 |
|  | *Yersinia* | 87 | 3.5 | 6.7 | 64.5 | 0.9 | 0.4 | 0.3 | 76.3 |
| fungi | *Alternaria* | 88 | 1.3 | 2.1 | 48.6 | 0.7 | 0.6 | 0.5 | 53.8 |
|  | *Aplosporella* | 89 | 1.6 | 1.7 | 70.1 | 0.8 | 0.6 | 0.3 | 75.1 |
|  | *Ascosphaera* | 90 | 2.4 | 2.5 | 67.9 | 1.2 | 1.9 | 1.2 | 77.1 |
|  | *Aspergillus* | 91 | 0.7 | 1.3 | 57.7 | 0.6 | 0.4 | 0.2 | 60.8 |
|  | *Aureobasidium* | 92 | 1.2 | 5.2 | 37.8 | 2.6 | 1.4 | 1.5 | 49.7 |
|  | *Blastomyces* | 93 | 1.4 | 3.5 | 69.5 | 1.8 | 0.8 | 0.4 | 77.4 |
|  | *Byssochlamys* | 94 | 1.7 | 1.4 | 69.6 | 0.8 | 0.8 | 0.3 | 74.6 |
|  | *Coccidioides* | 95 | 0.9 | 1.2 | 75.7 | 0.5 | 0.3 | 0.5 | 79.0 |
|  | *Fusarium* | 96 | 0.6 | 0.4 | 63.2 | 0.1 | 0.1 | 0.2 | 64.6 |
|  | *Histoplasma* | 97 | 0.5 | 0.5 | 73.9 | 0.3 | 0.1 | 0.1 | 75.5 |
|  | *Lasiodiplodia* | 98 | 1.6 | 3.3 | 37.2 | 1.2 | 0.8 | 1.6 | 45.7 |
|  | *Leptosphaeria* | 99 | 1.3 | 3.2 | 44.3 | 0.5 | 1.1 | 0.4 | 50.7 |
|  | *Macroventuria* | 100 | 2.3 | 3.9 | 57.5 | 0.8 | 0.6 | 0.4 | 65.4 |
|  | *Microsporum* | 101 | 1.0 | 1.3 | 69.8 | 0.4 | 0.7 | 0.2 | 73.4 |
|  | *Neurospora* | 102 | 2.8 | 4.4 | 57.1 | 1.1 | 0.7 | 0.8 | 66.9 |
|  | *Paracoccidioides* | 103 | 1.3 | 2.1 | 70.1 | 0.9 | 0.5 | 0.5 | 75.4 |
|  | *Penicilliopsis* | 104 | 1.9 | 2.6 | 65.6 | 1.5 | 0.6 | 0.6 | 72.9 |
|  | *Penicillium* | 105 | 0.6 | 0.8 | 55.0 | 2.2 | 0.6 | 0.2 | 59.3 |
|  | *Podospora* | 106 | 1.6 | 2.2 | 43.6 | 0.6 | 1.5 | 2.6 | 52.0 |
|  | *Talaromyces* | 107 | 0.6 | 0.3 | 67.1 | 0.1 | 0.1 | 0.1 | 68.3 |
|  | *Trichoderma* | 108 | 1.4 | 1.0 | 58.8 | 0.3 | 0.3 | 0.6 | 62.3 |
|  | *Trichophyton* | 109 | 1.1 | 1.3 | 72.1 | 0.6 | 0.3 | 0.3 | 75.8 |
|  | *Zygosaccharomyces* | 110 | 3.4 | 2.0 | 1.3 | 2.5 | 5.1 | 20.1 | 34.3 |
| viruses | *Myoviridae.undef* | 111 | 10.0 | 0.8 | 22.9 | 0.4 | 1.0 | 0.5 | 35.6 |
|  | *Siphoviridae.undef* | 112 | 8.3 | 1.0 | 6.6 | 0.9 | 2.0 | 1.0 | 19.8 |
|  | *Viruses.undef* |  |  |  |  |  |  |  |  |

Table S3. Order of taxa and the variance partitioning for each taxa as well as the R^2^ value as a measure of variance explained for the abundance model. The order of taxa is as shown in figures.

| Taxonomic group |  |  | fixed | total reads | random |  |  |  |  |
| --- | --- | --- | --- | --- | --- | --- | --- | --- | --- |
|  | Genus | order | time |  | sample | site | hive | beekeeper | R^2^ |
| plants | *Allium* | 1 | 6.7 | 4.1 | 4.3 | 5.3 | 2.9 | 6.1 | 29.5 |
|  | *Ananas* | 2 | 6.8 | 4.8 | 13.5 | 4.4 | 2.5 | 5.1 | 37.2 |
|  | *Arabidopsis* | 3 | 4.0 | 3.5 | 3.5 | 3.2 | 3.3 | 3.1 | 20.5 |
|  | *Brassica* | 4 | 3.1 | 3.6 | 6.4 | 6.0 | 4.9 | 8.9 | 32.9 |
|  | *Camellia* | 5 | 6.0 | 1.3 | 5.7 | 5.5 | 1.6 | 14.9 | 35.1 |
|  | *Capsella* | 6 | 6.2 | 2.8 | 4.9 | 4.7 | 11.3 | 7.2 | 37.1 |
|  | *Chamaenerion* | 7 | 6.1 | 6.5 | 6.1 | 5.5 | 4.0 | 5.4 | 33.5 |
|  | *Cicer* | 8 | 1.0 | 1.4 | 90.4 | 1.7 | 0.9 | 3.9 | 99.1 |
|  | *Corylus* | 9 | 3.4 | 2.7 | 2.3 | 5.2 | 2.6 | 17.2 | 33.3 |
|  | *Daucus* | 10 | 5.2 | 4.9 | 2.9 | 2.9 | 1.7 | 2.9 | 20.4 |
|  | *Dioscorea* | 11 | 2.6 | 1.8 | 4.9 | 2.2 | 1.7 | 2.1 | 15.3 |
|  | *Fagus* | 12 | 8.9 | 3.1 | 2.0 | 6.3 | 4.0 | 22.4 | 46.7 |
|  | *Fragaria* | 13 | 5.2 | 1.8 | 79.5 | 2.6 | 1.5 | 1.0 | 91.6 |
|  | *Glycine* | 14 | 2.9 | 1.4 | 75.3 | 3.3 | 1.5 | 4.2 | 88.7 |
|  | *Gossypium* | 15 | 5.4 | 2.1 | 10.0 | 6.3 | 3.2 | 22.4 | 49.2 |
|  | *Hordeum* | 16 | 3.9 | 3.2 | 2.4 | 2.2 | 1.3 | 1.5 | 14.5 |
|  | *Hypericum* | 17 | 5.8 | 4.0 | 5.9 | 3.5 | 3.1 | 3.5 | 25.8 |
|  | *Jasminum* | 18 | 4.5 | 6.5 | 11.1 | 6.2 | 2.5 | 18.3 | 49.0 |
|  | *Lactuca* | 19 | 24.3 | 4.8 | 6.0 | 10.4 | 7.6 | 14.0 | 67.0 |
|  | *Lathyrus* | 20 | 6.4 | 3.5 | 9.5 | 7.9 | 7.6 | 39.1 | 74.0 |
|  | *Lens* | 21 | 4.6 | 3.7 | 12.4 | 9.1 | 7.3 | 24.1 | 61.4 |
|  | *Lolium* | 22 | 2.8 | 2.8 | 1.7 | 1.9 | 0.9 | 1.0 | 11.2 |
|  | *Lotus* | 23 | 2.7 | 1.6 | 70.8 | 3.5 | 3.6 | 4.3 | 86.4 |
|  | *Lupinus* | 24 | 2.3 | 1.5 | 30.0 | 3.0 | 1.7 | 1.0 | 39.4 |
|  | *Malus* | 25 | 18.2 | 1.1 | 4.8 | 6.2 | 8.8 | 12.8 | 51.9 |
|  | *Medicago* | 26 | 1.0 | 1.6 | 86.3 | 2.3 | 1.2 | 4.7 | 97.1 |
|  | *Neobartsia* | 27 | 10.3 | 3.1 | 7.6 | 6.5 | 4.6 | 24.5 | 56.6 |
|  | *Panax* | 28 | 3.1 | 3.9 | 6.4 | 3.7 | 3.0 | 2.9 | 23.1 |
|  | *Papaver* | 29 | 5.0 | 1.6 | 2.6 | 3.0 | 2.1 | 4.1 | 18.5 |
|  | *Paraprenanthes* | 30 | 25.9 | 5.6 | 9.9 | 9.5 | 9.2 | 9.8 | 69.9 |
|  | *Picea* | 31 | 6.1 | 1.8 | 2.9 | 4.1 | 3.5 | 6.7 | 25.0 |
|  | *Pinus* | 32 | 10.0 | 1.2 | 2.7 | 2.9 | 1.4 | 3.4 | 21.6 |
|  | *Pisum* | 33 | 4.1 | 0.8 | 68.9 | 3.3 | 6.4 | 7.8 | 91.3 |
|  | *Populus* | 34 | 21.4 | 1.7 | 4.7 | 5.9 | 5.8 | 12.0 | 51.5 |
|  | *Prunus* | 35 | 17.5 | 0.6 | 41.5 | 3.7 | 4.9 | 3.2 | 71.4 |
|  | *Pyrus* | 36 | 7.6 | 1.9 | 5.4 | 5.1 | 1.8 | 12.9 | 34.7 |
|  | *Quercus* | 37 | 6.6 | 1.7 | 1.6 | 4.4 | 3.4 | 21.4 | 39.0 |
|  | *Rosa* | 38 | 5.6 | 0.8 | 86.4 | 2.1 | 0.9 | 1.9 | 97.7 |
|  | *Rubus* | 39 | 2.5 | 0.7 | 83.6 | 2.9 | 0.7 | 1.0 | 91.4 |
|  | *Salix* | 40 | 31.3 | 2.3 | 5.8 | 7.4 | 3.8 | 9.4 | 59.9 |
|  | *Solanum* | 41 | 4.4 | 5.6 | 4.0 | 5.2 | 1.9 | 10.1 | 31.2 |
|  | *Sorbus* | 42 | 9.8 | 3.7 | 3.3 | 6.0 | 4.2 | 17.9 | 44.9 |
|  | *Taraxacum* | 43 | 31.6 | 4.3 | 10.2 | 9.9 | 7.8 | 5.2 | 69.0 |
|  | *Theobroma* | 44 | 5.3 | 1.6 | 5.8 | 4.4 | 2.2 | 14.9 | 34.2 |
|  | *Trifolium* | 45 | 2.3 | 2.5 | 69.9 | 4.0 | 2.2 | 6.0 | 87.0 |
|  | *Triticum* | 46 | 4.1 | 2.3 | 2.4 | 2.5 | 1.5 | 1.6 | 14.3 |
|  | *Vaccinium* | 47 | 5.7 | 1.4 | 3.7 | 3.9 | 2.3 | 11.0 | 28.0 |
|  | *Vicia* | 48 | 3.5 | 2.1 | 13.9 | 7.3 | 19.0 | 34.9 | 80.6 |
|  | *Vitis* | 49 | 3.4 | 2.1 | 2.4 | 5.8 | 2.5 | 31.7 | 47.8 |
| bacteria | *Acetobacter* | 50 | 3.2 | 1.9 | 12.1 | 6.7 | 16.4 | 5.8 | 46.1 |
|  | *Acinetobacter* | 51 | 22.4 | 4.1 | 10.2 | 6.2 | 2.6 | 3.2 | 48.7 |
|  | *Amylolactobacillus* | 52 | 13.0 | 15.7 | 13.6 | 12.7 | 18.0 | 22.5 | 95.6 |
|  | *Arsenophonus* | 53 | 5.6 | 1.7 | 9.4 | 4.6 | 2.0 | 6.5 | 29.8 |
|  | *Asaia* | 54 | 7.0 | 4.4 | 9.3 | 5.7 | 8.6 | 6.9 | 42.0 |
|  | *Bacillus* | 55 | 4.1 | 3.4 | 44.0 | 2.9 | 1.7 | 2.1 | 58.2 |
|  | *Bartonella* | 56 | 4.6 | 5.0 | 11.3 | 5.5 | 2.7 | 21.2 | 50.2 |
|  | *Bifidobacterium* | 57 | 6.0 | 3.2 | 8.4 | 6.7 | 14.0 | 11.7 | 49.9 |
|  | *Bombella* | 58 | 3.9 | 1.9 | 25.0 | 4.4 | 9.1 | 1.8 | 46.1 |
|  | *Citrobacter* | 59 | 3.5 | 2.0 | 83.2 | 1.8 | 1.3 | 0.8 | 92.7 |
|  | *Commensalibacter* | 60 | 4.7 | 2.6 | 6.8 | 4.9 | 3.1 | 8.0 | 30.2 |
|  | *Companilactobacillus* | 61 | 4.1 | 2.5 | 3.4 | 3.8 | 2.6 | 4.9 | 21.3 |
|  | *Enterobacter* | 62 | 3.6 | 1.3 | 82.2 | 2.2 | 0.8 | 2.0 | 92.2 |
|  | *Enterococcus* | 63 | 3.8 | 3.2 | 10.2 | 5.1 | 4.7 | 7.7 | 34.7 |
|  | *Entomoplasma* | 64 | 9.8 | 1.4 | 12.1 | 8.1 | 7.3 | 22.7 | 61.5 |
|  | *Erwinia* | 65 | 5.5 | 5.9 | 56.3 | 2.9 | 1.5 | 3.4 | 75.6 |
|  | *Escherichia* | 66 | 1.7 | 1.6 | 80.8 | 3.3 | 1.3 | 1.1 | 89.8 |
|  | *Frischella* | 67 | 2.9 | 1.9 | 11.1 | 4.2 | 2.2 | 6.3 | 28.6 |
|  | *Gilliamella* | 68 | 3.4 | 2.0 | 10.0 | 4.1 | 1.8 | 4.7 | 26.0 |
|  | *Holzapfelia* | 69 | 10.8 | 12.0 | 37.2 | 9.8 | 13.7 | 16.6 | #### |
|  | *Klebsiella* | 70 | 3.3 | 1.3 | 87.8 | 1.5 | 0.4 | 2.1 | 96.5 |
|  | *Komagataeibacter* | 71 | 4.6 | 2.0 | 3.7 | 5.2 | 24.2 | 9.3 | 48.9 |
|  | *Lactiplantibacillus* | 72 | 5.3 | 4.2 | 13.9 | 6.5 | 5.4 | 13.5 | 48.8 |
|  | *Lactobacillus* | 73 | 2.5 | 1.3 | 7.9 | 2.8 | 1.6 | 3.1 | 19.2 |
|  | *Lactococcus* | 74 | 1.9 | 1.6 | 44.5 | 6.6 | 5.5 | 5.8 | 65.9 |
|  | *Listeria* | 75 | 11.0 | 10.1 | 32.8 | 8.4 | 12.1 | 8.8 | 83.2 |
|  | *Melissococcus* | 76 | 7.9 | 2.9 | 7.9 | 7.0 | 33.2 | 5.4 | 64.3 |
|  | *Mesoplasma* | 77 | 8.6 | 2.2 | 8.4 | 5.5 | 3.8 | 23.0 | 51.6 |
|  | *Orbus* | 78 | 7.9 | 4.9 | 52.0 | 6.9 | 5.7 | 4.2 | 81.6 |
|  | *Paenibacillus* | 79 | 12.1 | 6.0 | 8.3 | 12.2 | 10.8 | 25.1 | 74.4 |
|  | *Pantoea* | 80 | 1.2 | 0.6 | 67.6 | 6.0 | 0.9 | 15.9 | 92.3 |
|  | *Parasaccharibacter* | 81 | 3.2 | 1.8 | 29.4 | 3.3 | 8.2 | 1.4 | 47.3 |
|  | *Pectobacterium* | 82 | 1.5 | 2.9 | 86.0 | 1.3 | 0.4 | 0.8 | 93.0 |
|  | *Proteus* | 83 | 1.7 | 1.1 | 51.2 | 3.2 | 1.5 | 3.9 | 62.6 |
|  | *Pseudomonas* | 84 | 1.7 | 0.9 | 45.2 | 4.5 | 1.5 | 5.1 | 58.9 |
|  | *Rahnella* | 85 | 1.9 | 0.7 | 64.8 | 2.7 | 0.9 | 1.5 | 72.6 |
|  | *Salmonella* | 86 | 2.4 | 1.4 | 82.6 | 1.9 | 0.6 | 1.9 | 90.7 |
|  | *Serratia* | 87 | 4.2 | 2.3 | 74.5 | 1.7 | 0.5 | 2.6 | 85.9 |
|  | *Snodgrassella* | 88 | 3.4 | 2.4 | 2.9 | 3.5 | 2.7 | 4.5 | 19.5 |
|  | *Sodalis* | 89 | 4.6 | 11.2 | 9.4 | 5.1 | 3.1 | 8.3 | 41.9 |
|  | *Sphingomonas* | 90 | 7.2 | 2.9 | 5.3 | 5.4 | 6.0 | 5.3 | 32.1 |
|  | *Spiroplasma* | 91 | 8.5 | 3.3 | 6.5 | 4.1 | 3.3 | 3.1 | 28.8 |
|  | *Tatumella* | 92 | 2.9 | 2.3 | 52.7 | 5.0 | 1.3 | 2.4 | 66.6 |
|  | *Yersinia* | 93 | 3.2 | 3.2 | 77.1 | 2.4 | 0.6 | 0.8 | 87.2 |
| fungi | *Alternaria* | 94 | 14.0 | 6.2 | 6.8 | 6.7 | 60.0 | 3.0 | 96.7 |
|  | *Aplosporella* | 95 | 12.6 | 9.6 | 6.8 | 6.2 | 54.6 | 6.3 | 96.2 |
|  | *Ascosphaera* | 96 | 8.3 | 4.1 | 7.4 | 8.7 | 43.0 | 4.2 | 75.6 |
|  | *Aspergillus* | 97 | 7.3 | 5.1 | 2.5 | 8.4 | 55.1 | 7.2 | 85.5 |
|  | *Aureobasidium* | 98 | 9.4 | 5.6 | 5.1 | 9.7 | 57.8 | 3.4 | 91.0 |
|  | *Blastomyces* | 99 | 10.0 | 7.8 | 7.7 | 7.4 | 61.7 | 3.7 | 98.3 |
|  | *Byssochlamys* | 100 | 5.9 | 13.0 | 7.9 | 7.1 | 59.7 | 5.0 | 98.7 |
|  | *Coccidioides* | 101 | 11.1 | 4.0 | 12.2 | 4.7 | 64.8 | 1.7 | 98.4 |
|  | *Fusarium* | 102 | 9.3 | 3.2 | 4.5 | 6.4 | 56.6 | 3.4 | 83.2 |
|  | *Histoplasma* | 103 | 7.6 | 5.2 | 17.9 | 5.6 | 52.6 | 3.1 | 92.1 |
|  | *Lasiodiplodia* | 104 | 7.1 | 13.0 | 8.7 | 7.6 | 29.4 | 7.7 | 73.5 |
|  | *Leptosphaeria* | 105 | 14.8 | 3.4 | 3.1 | 7.6 | 48.6 | 10.3 | 87.8 |
|  | *Macroventuria* | 106 | 14.2 | 3.5 | 9.1 | 6.3 | 56.5 | 8.9 | 98.5 |
|  | *Microsporum* | 107 | 9.1 | 4.7 | 12.4 | 5.1 | 49.3 | 3.5 | 84.0 |
|  | *Neurospora* | 108 | 12.4 | 4.0 | 6.3 | 9.2 | 54.6 | 10.4 | 96.9 |
|  | *Paracoccidioides* | 109 | 6.6 | 6.4 | 6.7 | 5.9 | 68.5 | 2.0 | 96.1 |
|  | *Penicilliopsis* | 110 | 7.9 | 9.2 | 17.6 | 5.4 | 50.3 | 4.7 | 95.2 |
|  | *Penicillium* | 111 | 7.5 | 5.3 | 3.2 | 6.7 | 57.9 | 5.0 | 85.5 |
|  | *Podospora* | 112 | 6.8 | 11.1 | 7.0 | 6.4 | 56.5 | 3.7 | 91.5 |
|  | *Talaromyces* | 113 | 7.2 | 3.5 | 6.7 | 7.4 | 62.7 | 6.4 | 93.9 |
|  | *Trichoderma* | 114 | 16.2 | 2.5 | 6.5 | 8.4 | 56.2 | 7.8 | 97.6 |
|  | *Trichophyton* | 115 | 10.3 | 8.2 | 10.0 | 6.2 | 55.0 | 3.8 | 93.5 |
| viruses | *Myoviridae.undef* | 116 | 18.1 | 2.4 | 9.1 | 3.5 | 3.0 | 1.7 | 37.7 |
|  | *Siphoviridae.undef* | 117 | 13.3 | 4.5 | 2.0 | 4.4 | 5.7 | 3.4 | 33.3 |

*References for the supplementary material*

Achtman, M., Zurth, K., Morelli, G., Torrea, G., Guiyoule, A., & Carniel, E. (1999). Yersinia pestis, the cause of plague, is a recently emerged clone of Yersinia pseudotuberculosis. *Proceedings of the National Academy of Sciences of the United States of America*, *96*(24), 14043–14048. doi: 10.1073/PNAS.96.24.14043

Al-Fakih, A. A., Qasem, W., & Almaqtri, A. (2019). *Overview on antibacterial metabolites from terrestrial Aspergillus spp*. doi: 10.1080/21501203.2019.1604576

Allocati, N., Masulli, M., Alexeyev, M. F., & Ilio, C. Di. (2013). Escherichia coli in Europe: An Overview. *OPEN ACCESS Int. J. Environ. Res. Public Health*, *10*, 10. doi: 10.3390/ijerph10126235

Aneke, C. I., Otranto, D., & Cafarchia, C. (2018). Therapy and Antifungal Susceptibility Profile of Microsporum canis. *Journal of Fungi*, *4*, 107. doi: 10.3390/jof4030107

Baby, V., Lachance, J.-C., Gagnon, J., Lucier, J.-F., Matteau, D., Knight, T., & Rodrigue, S. (2018). Inferring the Minimal Genome of Mesoplasma florum by Comparative Genomics and Transposon Mutagenesis . *MSystems*, *3*(3). doi: 10.1128/MSYSTEMS.00198-17

Bagal, U. R., Ireland, M., Gross, A., Fischer, J., Bentz, M., Berkow, E. L., … Chow, N. A. (2022). Molecular Epidemiology of of Blastomyces gilchristii Clusters, Minnesota, USA. *Emerging Infectious Diseases*, *28*(9), 1924–1926.

Borges-Walmsley, M. I., Chen, D., Shu, X., & Walmsley, A. R. (2002). The pathobiology of Paracoccidioides brasiliensis. *Trends in Microbiology*, *10*(2), 80–87. doi: 10.1016/S0966-842X(01)02292-2

Budge, G. E., Adams, I., Thwaites, R., Pietravalle, S., Drew, G. C., Hurst, G. D. D., … Brown, M. (2016). Identifying bacterial predictors of honey bee health. *Journal of Invertebrate Pathology*, *141*, 41–44. doi: 10.1016/j.jip.2016.11.003

Carina Audisio, M., Torres, M. J., Sabaté, D. C., Ibarguren, C., & Apella, M. C. (2011). Properties of different lactic acid bacteria isolated from Apis mellifera L. bee-gut. *Microbiological Research*, *166*(1), 1–13. doi: 10.1016/J.MICRES.2010.01.003

Contreras-Cornejo, H. A., Macías-Rodríguez, L., Cortés-Penagos, C., & Ló Pez-Bucio, J. (n.d.). *Trichoderma virens, a Plant Beneficial Fungus, Enhances Biomass Production and Promotes Lateral Root Growth through an Auxin-Dependent Mechanism in Arabidopsis 1[C][W][OA]*. doi: 10.1104/pp.108.130369

Corby-Harris, V., Snyder, L., Meador, C., Naldo, R., Mott, B. M., & Anderson, K. E. (2016). Parasaccharibacter apium, gen. nov., sp. nov., Improves Honey Bee (Hymenoptera: Apidae) Resistance to Nosema. *Journal of Economic Entomology*, *109*(2), 537–543. doi: 10.1093/JEE/TOW012

Corby-Harris, V., Snyder, L., Schwan, M. R., Maes, P., McFrederick, Q. S., & Anderson, K. E. (2014). Origin and effect of Alpha 2.2 Acetobacteraceae in honey bee larvae and description of Parasaccharibacter apium gen. nov., sp. nov. *Applied and Environmental Microbiology*, *80*(24), 7460–7472. doi: 10.1128/AEM.02043-14

Czajkowski, R., Pé, M. C. M., Van Veen, J. A., & Van Der Wolf, J. M. (n.d.). *Control of blackleg and tuber soft rot of potato caused by Pectobacterium and Dickeya species: a review*. doi: 10.1111/j.1365-3059.2011.02470.x

Dalcin, D., Rothstein, A., Spinato, J., Escott, N., & Kus, J. V. (2016). Blastomyces gilchristii as Cause of Fatal Acute Respiratory Distress Syndrome. *Emerging Infectious Diseases*, *22*(2), 22–24.

Debbarma, R., Kamil, D., Maya Bashyal, B., Choudhary, S. P., & Thokla, P. (2021). First report of root rot disease on Solanum lycopersicum L. caused by Fusarium vanettenii in India. *Journal of Phytopathology*, *169*(11–12), 752–756. doi: 10.1111/JPH.13047

Dong, Z. X., Li, H. Y., Chen, Y. F., Wang, F., Deng, X. Y., Lin, L. B., … Guo, J. (2020). Colonization of the gut microbiota of honey bee (Apis mellifera) workers at different developmental stages. *Microbiological Research*, *231*, 126370. doi: 10.1016/J.MICRES.2019.126370

Engel, P., Martinson, V. G., & Moran, N. A. (2012). Functional diversity within the simple gut microbiota of the honey bee. *Proceedings of the National Academy of Sciences of the United States of America*, *109*(27), 11002–11007. doi: 10.1073/PNAS.1202970109/SUPPL_FILE/SD01.XLS

Erban, T., Ledvinka, O., Kamler, M., Nesvorna, M., Hortova, B., Tyl, J., … Hubert, J. (2017). Honeybee (Apis mellifera)-associated bacterial community affected by American foulbrood: detection of Paenibacillus larvae via microbiome analysis. *Scientific Reports 2017 7:1*, *7*(1), 1–10. doi: 10.1038/s41598-017-05076-8

Escalante-Pérez, M., Jaborsky, M., Lautner, S., Fromm, J., Müller, T., Dittrich, M., … Ache, P. (2012). Poplar Extrafloral Nectaries: Two Types, Two Strategies of Indirect Defenses against Herbivores 1[C][W]. *Plant Physiology*, *159*, 1179–1191. doi: 10.1104/pp.112.196014

Evison, S. E., & Jensen, A. B. (2018, April 1). The biology and prevalence of fungal diseases in managed and wild bees. *Current Opinion in Insect Science*, Vol. 26, pp. 105–113. Elsevier Inc. doi: 10.1016/j.cois.2018.02.010

Foley, K., Fazio, G., Jensen, A. B., & Hughes, W. O. H. (2014a). The distribution of Aspergillus spp. opportunistic parasites in hives and their pathogenicity to honey bees. *Veterinary Microbiology*, *169*(3–4), 203–210. doi: 10.1016/J.VETMIC.2013.11.029

Foley, K., Fazio, G., Jensen, A. B., & Hughes, W. O. H. (2014b). The distribution of Aspergillus spp. opportunistic parasites in hives and their pathogenicity to honey bees. *Veterinary Microbiology*, *169*(3–4), 203–210. doi: 10.1016/J.VETMIC.2013.11.029

Fünfhaus, A., Ebeling, J., & Genersch, E. (2018, April 1). Bacterial pathogens of bees. *Current Opinion in Insect Science*, Vol. 26, pp. 89–96. Elsevier Inc. doi: 10.1016/j.cois.2018.02.008

Gasparich, G. E. (2014). 38 The Family Entomoplasmataceae. In E. Thompson, E. F. Rosenberg, S. De Long, E. Lory, & F. Stackebrandt (Eds.), *The Prokaryotes* (pp. 505–514). doi: 10.1007/978-3-642-30120-9_390

Hamon, M., Bierne, H., & Cossart, P. (2006). Listeria monocytogenes: a multifaceted model. *NATURE REVIEWS | MICROBIOLOGY*, *4*, 423. doi: 10.1038/nrmicro1413

Hartmann, U., Forsgren, E., Charrière, J. D., Neumann, P., & Gauthier, L. (2015). Dynamics of Apis mellifera filamentous virus (AmFV) infections in honey bees and relationships with other parasites. *Viruses*, *7*(5), 2654–2667. doi: 10.3390/v7052654

Huang, H. C., Phillippe, L. M., & Phillippe, R. C. (1990). Pink seed of pea: a new disease caused by Erwinia rhapontici. *Canadian Journal of Plant Pathology*, *12*, 445–448. doi: 10.1080/07060669009500990

Hubert, J., Erban, T., Kamler, M., Kopecky, J., Nesvorna, M., Hejdankova, S., … Zurek, L. (2015). Bacteria detected in the honeybee parasitic mite Varroa destructor collected from beehive winter debris. *Journal of Applied Microbiology*, *119*(3), 640–654. doi: 10.1111/JAM.12899

Iorizzo, M., Testa, B., Ganassi, S., Lombardi, S. J., Ianiro, M., Letizia, F., … Geddes-Mcalister, J. (2021). Probiotic Properties and Potentiality of Lactiplantibacillus plantarum Strains for the Biological Control of Chalkbrood Disease. *J. Fungi*, *7*. doi: 10.3390/jof7050379

Johnson, K. B., Stockwell, V. O., Burgett, D. M., Sugar, D., & Loper, J. E. (1993). Dispersal of Erwinia amylovora and Pseudomonas fluorescens by honey bees from hives to apple and pear blossoms. *Phytopathology*, *83*, 478–484.

Kačániová, M., Gasper, J., & Terentjeva, M. (2019). Antagonistic effect of gut microbiota of honeybee (Apis mellifera) against causative agent of American Foulbrood Paenibacillus larvae. *Journal of Microbiology, Biotechnology and Food Sciences*, 478–481. doi: 10.15414/jmbfs.2019.9.special.478-481

Kaper, J. B., Nataro, J. P., & Mobley, H. L. T. (2004). Pathogenic Escherichia coli. *Nature Reviews Microbiology 2004 2:2*, *2*(2), 123–140. doi: 10.1038/nrmicro818

Keller, A., McFrederick, Q. S., Dharampal, P., Steffan, S., Danforth, B. N., & Leonhardt, S. D. (2021, April 1). (More than) Hitchhikers through the network: The shared microbiome of bees and flowers. *Current Opinion in Insect Science*, Vol. 44, pp. 8–15. Elsevier Inc. doi: 10.1016/j.cois.2020.09.007

Kešnerová, L., Moritz, R., & Engel, P. (2016). Bartonella apis sp. nov., a honey bee gut symbiont of the class Alphaproteobacteria. *International Journal of Systematic and Evolutionary Microbiology*, *66*(1), 414–421. doi: 10.1099/IJSEM.0.000736

Khan, S., Somerville, D., Frese, M., & Nayudu, M. (2020). Environmental gut bacteria in European honey bees (Apis mellifera) from Australia and their relationship to the chalkbrood disease. *PLoS ONE*, *15*(8 august), e0238252. doi: 10.1371/journal.pone.0238252

Kim, P. S., Shin, N.-R., Kim, J. Y., Yun, J.-H., Hyun, D.-W., & Bae, J.-W. (2014). Acinetobacter apis sp. nov., isolated from the intestinal tract of a honey bee, Apis mellifera. *Journal of Microbiology 2014 52:8*, *52*(8), 639–645. doi: 10.1007/S12275-014-4078-0

Knodler, L. A., & Elfenbein, J. R. (2019). Salmonella enterica. *Trends in Microbiology*, *27*(11). doi: 10.1016/j.tim.2019.05.002

Kollath, D. R., Miller, K. J., & Barker, B. M. (2019). *The mysterious desert dwellers: Coccidioides immitis and Coccidioides posadasii, causative fungal agents of coccidioidomycosis*. doi: 10.1080/21505594.2019.1589363

Kumar, G., Menanteau-Ledouble, S., Saleh, M., & El-Matbouli, M. (2015). Yersinia ruckeri, the causative agent of enteric redmouth disease in fish. *Veterinary Research*, *46*(1), 1–10. doi: 10.1186/S13567-015-0238-4/TABLES/1

Kurtzman, C. P. (1990). DNA Relatedness among Species of the Genus Zygosaccharomyces. *YEAST*, *6*, 19.

Kutcher, H. R., Keri, M., McLaren, D. L., & Rimmer, S. R. (2007). Pathogenic variability of Leptosphaeria maculans in western Canada. *Canadian Journal of Plant Pathology*, *29*(4), 388–393. doi: 10.1080/07060660709507484

Larsson, J. (2022). eulerr: Area-Proportional Euler and Venn Diagrams with Ellipses [R package eulerr version 6.1.0]. doi: //CRAN.R-project.org/package=eulerr

Liu, G., Tao, C., Zhu, B., Bai, W., Zhang, L., Wang, Z., & Liang, X. (2016). Identification of Zygosaccharomyces mellis strains in stored honey and their stress tolerance. *Food Science and Biotechnology 2016 25:6*, *25*(6), 1645–1650. doi: 10.1007/S10068-016-0253-X

Liu, L. H., Wang, N. Y., Wu, A. Y. J., Lin, C. C., Lee, C. M., & Liu, C. P. (2018). Citrobacter freundii bacteremia: Risk factors of mortality and prevalence of resistance genes. *Journal of Microbiology, Immunology and Infection*, *51*(4), 565–572. doi: 10.1016/J.JMII.2016.08.016

Loncaric, I., Heigl, H., Licek, E., Moosbeckhofer, R., Busse, H. J., & Rosengarten, R. (2009). Typing of Pantoea agglomerans isolated from colonies of honey bees (Apis mellifera) and culturability of selected strains from honey. *Apidologie*, *40*(1), 40–54. doi: 10.1051/apido/2008062

Ma, L. J., Geiser, D. M., Proctor, R. H., Rooney, A. P., O’Donnell, K., Trail, F., … Kazan, K. (2013). Fusarium pathogenomics. *Annual Review of Microbiology*, *67*, 399–416. doi: 10.1146/annurev-micro-092412-155650

McCoy, R. E., Basham, H. G., & Tully, J. G. (1984). Acholeplasma florum, a new species isolated from plants. *International Journal of Systematic Bacteriology*, *34*(1), 11–15. doi: 10.1099/00207713-34-1-11/CITE/REFWORKS

Mittal, J., Ponce, M. G., Gendlina, I., & Nosanchuk, J. D. (2019). Histoplasma capsulatum: Mechanisms for pathogenesis. *Current Topics in Microbiology and Immunology*, *422*, 157–191. doi: 10.1007/82_2018_114/COVER

Nowak, A., Szczuka, D., Górczyńska, A., Motyl, I., & Kręgiel, D. (2021). Characterization of apis mellifera gastrointestinal microbiota and lactic acid bacteria for honeybee protection—a review. *Cells*, *10*(3), 1–29. doi: 10.3390/CELLS10030701

Oksanen, J., Blanchet, F. G., Friendly, M., Kindt, R., Legendre, P., Mcglinn, D., … Maintainer, H. W. (2019). *Package ‘vegan’ Title Community Ecology Package Version 2.5-6*.

Olofsson, T. C., Alsterfjord, M., Nilson, B., Butler, È., & Vásquez, A. (2014). Lactobacillus apinorum sp. nov., Lactobacillus mellifer sp. nov., Lactobacillus mellis sp. nov., Lactobacillus melliventris sp. nov., Lactobacillus kimbladii sp. nov., Lactobacillus helsingborgensis sp. nov. and lactobacillus kullabergensis sp. nov., isolated from the honey stomach of the honeybee Apis mellifera. *International Journal of Systematic and Evolutionary Microbiology*, *64*, 3109–3119. doi: 10.1099/ijs.0.059600-0

Olofsson, T. C., & Vásquez, A. (2008). Detection and identification of a novel lactic acid bacterial flora within the honey stomach of the honeybee Apis mellifera. *Current Microbiology*, *57*(4), 356–363. doi: 10.1007/s00284-008-9202-0

Ostaff, D. P., Mosseler, A., Johns, R. C., Javorek, S., Klymko, J., & Ascher, J. S. (2015). Willows (salix spp.) as pollen and nectar sources for sustaining fruit and berry pollinating insects. *Canadian Journal of Plant Science*, *95*(3), 505–516. doi: 10.4141/CJPS-2014-339/ASSET/IMAGES/CJPS-2014-339TAB4.GIF

Papini, R., Nardoni, S., Fanelli, A., & Mancianti, F. (2009). High Infection Rate of Trichophyton verrucosum in Calves from Central Italy. *Zoonoses and Public Health*, *56*(2), 59–64. doi: 10.1111/J.1863-2378.2008.01157.X

Piva, S., Giacometti, F., Marti, E., Massella, E., Cabbri, R., Galuppi, R., & Serraino, A. (2020). Could honey bees signal the spread of antimicrobial resistance in the environment? *Letters in Applied Microbiology*, *70*(5), 349–355. doi: 10.1111/lam.13288

Quintana, S., Brasesco, C., Porrini, L. P., Di Gerónimo, V., Eguaras, M. J., & Maggi, M. (2019). First molecular detection of Apis mellifera filamentous virus (AmFV) in honey bees (Apis mellifera) in Argentina. *Journal of Apicultural Research*, *60*(1), 111–114. doi: 10.1080/00218839.2019.1690100

Raymann, K., & Moran, N. A. (2018, April 1). The role of the gut microbiome in health and disease of adult honey bee workers. *Current Opinion in Insect Science*, Vol. 26, pp. 97–104. Elsevier Inc. doi: 10.1016/j.cois.2018.02.012

Salvatore, M. M., Andolfi, A., & Nicoletti, R. (2020). The Thin Line between Pathogenicity and Endophytism: The Case of Lasiodiplodia theobromae. *Agriculture*, *10*, 488. doi: 10.3390/agriculture10100488

Shoreit, M. N., & Bagy, M. M. K. (1995). Mycoflora associated with stonebrood disease in honeybee colonies in Egypt. *Microbiological Research*, *150*(2), 207–211. doi: 10.1016/S0944-5013(11)80058-3

Smith, E. A., Anderson, K. E., Corby-Harris, V., McFrederick, Q. S., & Newton, I. L. G. (2020, May 8). Reclassification of seven honey bee symbiont strains as Bombella apis. *BioRxiv*, p. 2020.05.06.081802. bioRxiv. doi: 10.1101/2020.05.06.081802

Tsuge, T., Harimoto, Y., Akimitsu, K., Ohtani, K., Kodama, M., Akagi, Y., … Otani, H. (2013). Host-selective toxins produced by the plant pathogenic fungus Alternaria alternata. *FEMS Microbiology Reviews*, *37*, 44–46. doi: 10.1111/j.1574-6976.2012.00350.x

Wheat, L. J., Byron, E., Batteiger, M. D., & Boonmee Sathapatayavongs, M. D. (1990). Histoplasma capsulatum infections of the central nervous system A clinical review. *Nucl. Phys.*, *69*(4), 244.

Yañez, O., Gauthier, L., Chantawannakul, P., & Neumann, P. (2016). Endosymbiotic bacteria in honey bees: Arsenophonus spp. are not transmitted transovarially. *FEMS Microbiology Letters*, *363*(14). doi: 10.1093/femsle/fnw147
